# Perceptions of simulated artificial intelligence in medical consultations: associations with stress, memory, and perceived credibility

**DOI:** 10.1038/s41746-026-03022-5

**Published:** 2026-07-29

**Authors:** Carlotta J. Mayer, Tristan L. Swysen, Tobias R. Kurz, Tobias I. Stephan, Daniela Geisel, Elena S. Doll, Nassim Mahal, Seraina P. Lerch, Sebastian Sailer, Christian P. Schaaf, Christian J. Merz, Torsten Wüstenberg, Johannes C. Ehrenthal, Steffen Walter, Julia Mahal, Beate Ditzen

**Affiliations:** 1 Laboratory for Clinical Neuropsychology, Department of Psychology, Heidelberg University, Heidelberg, Germany; 2 Institute of Medical Psychology, Heidelberg University Hospital, Heidelberg, Germany; 3 Heidelberg University, Heidelberg, Germany; 4 Clinic of Psychosomatic and Psychotherapy, Department of Medical Psychology, University Hospital Ulm, Ulm, Germany; 5 Department of Medical Psychology, University Medicine Greifswald, Greifswald, Germany; 6 Institute of Human Genetics, Heidelberg University, Medical Faculty Heidelberg, Heidelberg, Germany; 7 German Centre for Mental Health, Partner Site Heidelberg-Mannheim-Ulm, Mannheim, Germany; 8 Department of Cognitive Psychology, Institute of Cognitive Neuroscience, Faculty of Psychology, Ruhr University Bochum, Bochum, Germany; 9 Core Facility for Neurocognitive Systems Research (CNSR), Heidelberg University, Heidelberg, Germany; 10 Department of Psychology, University of Cologne, Cologne, Germany; 11 Clinical Biopsychology and Psychotherapy, Department of Psychology, University of Zurich, Zurich, Switzerland

**Keywords:** Computational biology and bioinformatics, Health care, Mathematics and computing, Psychology, Psychology

## Abstract

The growing integration of digital technologies into healthcare requires understanding the impact of different consultation modalities on patient stress, memory and perceived credibility. This study compared different consultation modalities (human physician, in person or via video call; AI-style physician, as a chatbot or avatar) in standardized simulated medical consultations involving the delivery of bad news. For the AI-styled physician consultations, a Wizard of Oz design was used, in which participants were told they interacted with an AI physician while a human controlled the interaction. 163 healthy participants experienced either a human or an AI physician. Stress was measured through ratings and salivary cortisol, alongside memory retrieval and situation credibility. Human-based consultations led to higher stress levels than AI-styled formats. Greater perceived credibility was associated with stronger stress responses. Memory retrieval was lowest in the AI-styled chatbot condition. These findings show that different consultation modalities are associated with varying levels of stress and memory in medical settings. AI-styled physician interactions may reduce stress in routine medical communication, but their use should be carefully considered for critical medical communication.

## Introduction

Digitalization and artificial intelligence (AI) are increasingly influencing medical consultations, for example, through decision support, patient monitoring, or remote consultations^1^. These technological advancements offer new opportunities: Complex and multimodal data make precise diagnoses and customized treatments possible^2^. At the same time, it creates challenges such as the limited safety of conversational agents^3^ and physicians’ new role as translators of technological information for patients^4^. Successful clinical integration of these systems, therefore, requires consideration of ethical challenges such as data protection and patient safety^2^.

A key aspect of consultations is the delivery of bad news, which is a stressful experience for both physicians and patients^5^. Effective communication of bad news requires stress management while ensuring the clear and accurate delivery of critical information, ensuring that patients understand and remember what is communicated. Emotional stress during such interactions can impair memory retrieval, potentially affecting patient comprehension and adherence to medical recommendations^6^. As digital and AI-supported consultation modalities continue to be explored in healthcare settings^7–9^, concerns arise about their effectiveness and the psychological and physiological demands they may place on patients and physicians.

Regardless of the degree of technological integration, medical consultations place a considerable burden on both physicians and patients, with patients often experiencing elevated stress levels even before the specific reason for the consultation is discussed^10,11^. This highlights the importance of designing medical encounters to minimize additional stress on patients. Notably, physiological arousal fluctuates across different phases of medical consultations: Patients’ arousal decreases during the initial news phase, suggesting that anticipatory stress may decline quickly once the consultation begins. During the subsequent information phase, arousal remains steady, but responses vary depending on the type of news delivered^10^. Beyond the content itself, the manner in which information is communicated is critical. Physician behavior during consultations plays a key role in alleviating patient stress. In particular, communication styles that are facilitative, patient-centered, and empathic have been associated with reduced physiological stress responses and improved patient outcomes^12,13^. Such styles include acknowledging patients’ concerns, providing clear and structured information, and supporting patients in processing the situation. These behaviors provide both emotional support, such as empathy and attentive listening, and informational support (e.g., helping patients understand and evaluate medical information), which are central to effective stress regulation in medical interactions. Self-reported anxiety and feelings of loss of control can lead patients to avoid medical consultations altogether, underscoring the need for a supportive and patient-centered consultation environment^14^. These findings align with experimental research showing that social support can reduce physiological stress responses during acute stress^15–17^.

Given the importance of supportive interactions in mitigating patient stress, and the emerging potential for AI-based systems to facilitate such interactions, it is relevant to explore which aspects of medical consultation can be supported technologically – for example, through conversational agents. These AI-driven systems appear as chatbots for written text interactions or as avatars for speech, video, and image-based exchanges to enable real-time interaction and are already deployed in patient education, mental health, chronic disease monitoring, and early diagnostics^18,19^.

Current studies show broad acceptance of digital formats, especially among younger, digitally literate patients. Video consultations can be perceived as equally empathic as in-person visits^20^, and satisfaction with text-based communication increases when responses are detailed and emotionally supportive^21^. Acceptance depends on perceived quality, efficiency, and trust^22^. Patients tend to prefer conversational agents for less complex or more stigmatized topics such as mental or sexual health, where anonymity is valued^23^. In contrast, human physicians remain the preferred option for sensitive or diagnostically uncertain situations^24,25^.

Digital formats shape communication expectations: Patients often seek factual information in online consultations, while relational aspects tend to dominate in face-to-face settings^26^. Some studies suggest that satisfaction and perceived relational quality may not differ substantially across digital and in-person consultations. Emotional safety appears to be a key condition for meaningful engagement^27^, and features such as virtual embodiment can enhance emotional relevance and perceived presence^28^. These findings indicate that human-like AI interfaces could support relational aspects of care. Nevertheless, technological barriers and unequal access remain persistent challenges that may reinforce existing health disparities^29^.

Although technological development is advancing rapidly, empirical research on its clinical implementation and impact remains limited. Acceptance of AI-assisted consultations varies by age, prior knowledge, and digital experience^30^. Clinicians also note that certain conditions or patient characteristics may be less suited for remote or AI-based interactions^31^. Emerging evidence suggests that acceptance of AI-supported tools is closely tied to patients’ trust in their physician and concerns regarding data security: patients report lower comfort and altered communication behavior, such as self-censoring, when uncertainty about data use persists, whereas trust in the physician can mitigate these concerns and increase willingness to engage with the technology^32^. Further research is needed to clarify where and for whom conversational AI can meaningfully support – not replace – human care.

Li^33^ identified key stress-related barriers to acceptance of medical AI systems, including algorithm aversion, robophobia, loss of perceived human care, and doubts regarding AI’s applicability to rare or complex conditions. In contrast, evidence from real physician-patient chats suggests that digital communication can still be experienced as empathic and symptom-relieving—provided it is carried out by human professionals with appropriate sensitivity^34^. However, the stress-reducing potential of remote interaction may be limited in certain contexts: for instance, during the COVID-19 pandemic, Tokumasu et al.^35^ found that only offline communication was associated with decreased stress levels in medical students, while online formats showed no significant effect. When comparing human physicians across different consultation modalities, studies suggest that in-person consultations are experienced as more emotionally intense, whereas remote formats can reduce subjective stress^26^. However, when the interaction partner is an AI system, additional factors come into play. The development of trust in AI interaction partners in organizational contexts is shaped by both cognitive factors, such as reliability and transparency, and emotional factors, including affective reactions and perceived emotional closeness^36^. These findings underscore the importance of context, communication quality, and user expectations when evaluating the emotional impact of digital medical consultations. Therefore, there is a need to empirically test AI-physician interactions in real clinical contexts rather than assuming generalizability across medical domains.

Patients often struggle to retain medical information, particularly when the content is complex or poorly structured. On average, they forget 40–80% of medical information immediately after a consultation, and nearly half of what is remembered may be inaccurate^6^. Perceived stress during consultations impairs memory, although this relationship depends on the timing and regulation of the stress response. Experimental work suggests that stress enhances encoding when it occurs shortly before presentation of the information to memorize, during, or immediately after the encoding phase. In contrast, stress impairs memory encoding when it precedes the encoding phase by a longer interval – likely due to different cortisol activity phases^37^. This implies that physiological stress markers, such as salivary cortisol, should be assessed dynamically across time points.

Evidence from physician-patient interactions remains mixed. One study found no mediation between physician communication and memory via physiological arousal, although heart rate moderated retrieval indirectly^38^. Similarly, no direct associations between subjective and physiological stress measures and memory performance were reported in a subsequent study^39^. In contrast, affective physician communication has been shown to enhance retrieval and reduce anxiety, even if stress reduction did not fully mediate memory effects^39,40^. Furthermore, consistent eye contact during oncology consultations significantly improved patients’ recognition performance^40^. Taken together, these findings suggest that while stress can affect memory under certain conditions, supportive communication consistently facilitates retrieval—possibly through emotional buffering.

While prior studies on medical consultations have largely focused on human-to-human interaction, research on AI-based agents in healthcare has primarily addressed usability and acceptance—leaving emotional, cognitive, and physiological outcomes underexplored. To address this gap, the present study examined subjective and physiological stress responses, as well as memory retrieval, in a simulated diagnostic consultation involving bad news delivery. Rather than making claims about AI as a technology class, this study focuses on specific, controlled configurations of AI-styled physician interactions within a simulated clinical setting. In the following, “AI*” refers exclusively to interactions with the simulated AI agents used in this study. Importantly, the observed effects reflect the participants’ perceptions and expectations of interacting with an entity believed to be AI; the interaction was deliberately simulated, and the study did not aim to approximate realism in terms of actual AI capabilities.

Participants were randomly assigned to one of four conditions reflecting different consultation modalities (human physician in-person, human physician via video visit, AI* physician communicating via a text-based chatbot, and AI* physician represented by a virtual avatar). This design enables a systematic comparison of interactions with simulated AI* physician configurations and human physicians in emotionally salient contexts.

The study first investigated whether consultation modality would influence subjective stress and physiological arousal (salivary cortisol): Based on prior research pointing to barriers of AI-mediated consultations^33^ and prior findings on stress reduction in offline communication^35^, we hypothesized that AI*-physician conditions would induce higher stress compared to human-physician interactions (H1). Second, we hypothesized that consultation modality affects patients’ memory retrieval for medical information (H2). This is grounded in findings indicating that interpersonal and affective elements of medical communication directly facilitate patients’ memory retrieval^40^, and that nonverbal cues enhance attentional focus and encoding processes^41^. Based on previous findings suggesting that physiological stress may influence memory retrieval^37,42–44^, we also hypothesized that cortisol concentrations would moderate the effect of consultation modality on patients’ memory retrieval. Based on the evidence outlined above^33,35,37,40–44^, we hypothesized that AI*-physician conditions would induce higher stress and reduce memory retrieval compared to human-physician interactions. Exploratory analyses further examined the role of perceived credibility of the situation modulating these outcomes.

## Results

### Subjective stress (*H1*)

**Fig. 1:**
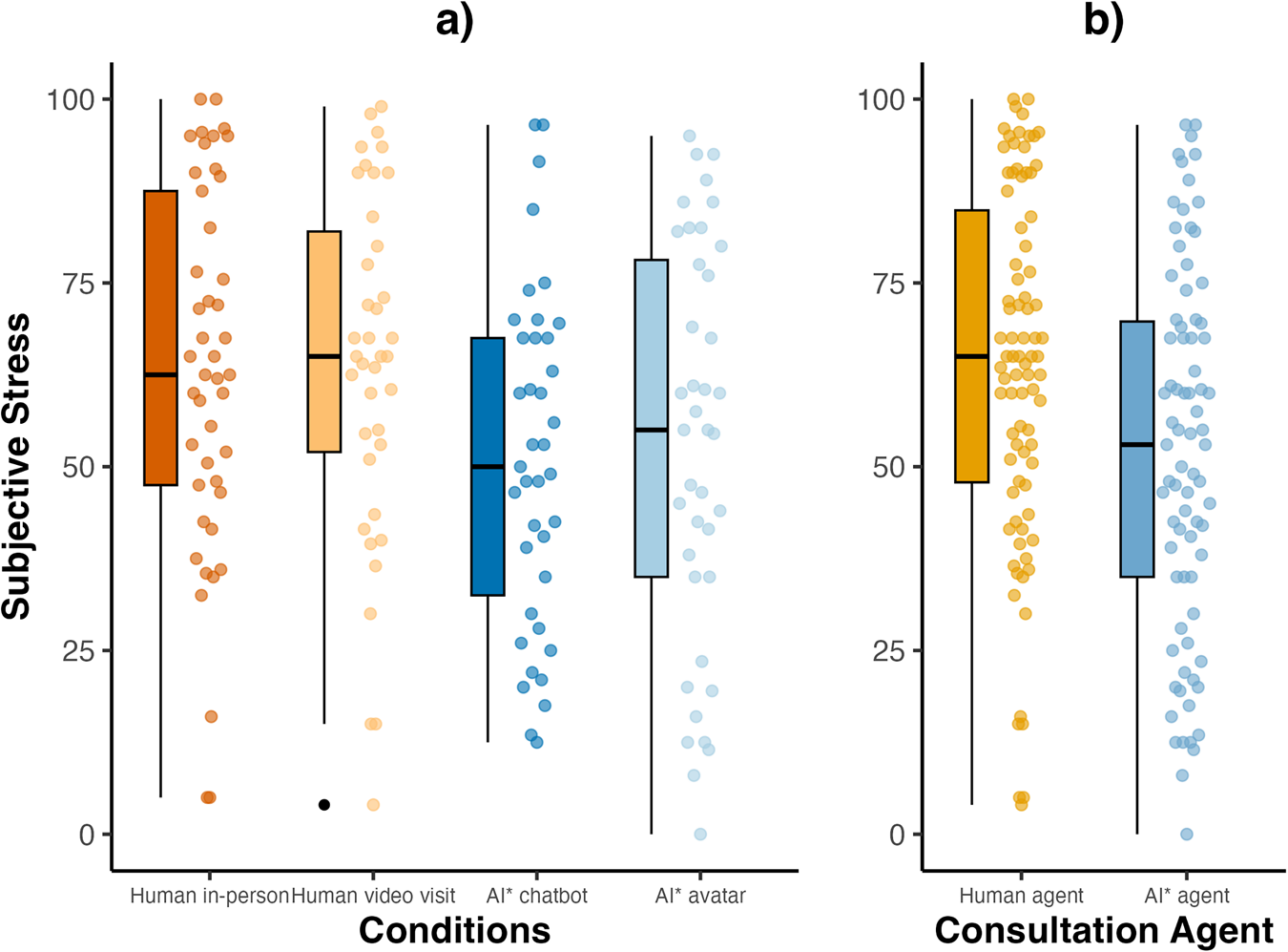
Subjective stress across consultation modalities. Panel **a** shows subjective stress levels across all four consultation modalities. Panel **b** shows subjective stress levels across two combined agent conditions. All orange boxplots and dots represent human physician conditions; all blue boxplots and dots represent AI physician conditions.

A one-way ANOVA was conducted to examine the effect of all four conditions on subjective stress. The ANOVA revealed a statistically significant main effect of condition on subjective stress (*F*(3, 159) = 2.91, *p* = .036, *η²* = .05). Post-hoc comparisons using Tukey’s HSD test showed no significant differences between the human in-person (*M* = 63.31, SD = 25.04), human video visit (*M* = 63.72, SD = 23.98), AI* chatbot (*M* = 51.08, SD = 22.97), and AI* avatar conditions (*M* = 53.00, SD = 27.33), with all *p* > .05. This analysis was repeated for combined agent groups *human* versus AI*; with human agents combining in-person and video visit and AI* agents containing AI* chatbot and AI* avatar. A Wilcoxon Rank test showed significantly higher levels of subjective stress in the human condition (*M* = 63.50, SD = 24.41) than in the AI* condition (*M* = 52.05, SD = 25.13, *W* = 4182, *p* = .004, *d* = −.46). Figure 1a shows the data distributions of all four conditions; Fig. 1b depicts the distribution of the two groups.

### sCort (*H1*)

Cortisol concentrations varied substantially across individuals, with a smaller proportion of variance explained by within-person fluctuations over time (intraclass correlation coefficient for null model, ICC = 0.803). Therefore, associations between within- and between-person variations in sCort and communication format were subsequently examined using mixed-effects models. The intraclass correlation coefficient (ICC) was 0.917, indicating a high degree of clustering at the subject level and substantial between-subject variability in baseline cortisol levels and time slopes. The whole model explained 90.9% of the variance in sCort (Pseudo-*R*²_total_ = 0.909), with a variance proportion explained by fixed effects of 14.6% (Pseudo-*R*²_fixed_ = 0.146). Cook’s distance method identified 56 data points as outliers. Visual check of data points over time was unremarkable, and sensitivity analysis excluding all detected outliers showed the same effects as the model including all cases (see Supplementary Materials E)).

A significant main effect of time was identified, indicating that log-transformed sCort concentrations decreased linearly over time (*β* = –0.086, *p* < .001). No significant main effects were observed for the four conditions. A significant interaction between time and consultation modality was observed for the AI* avatar condition, indicating a stronger decline in sCort over time compared to the human in-person condition (*β* = –0.042, *p* = .046). No significant interactions between time and consultation modality were found for AI* chatbot (*p* = .170) or human video visit (*p* = .713). More detailed information is depicted in Table 1.

**Table 1:** sCort concentrations across consultation modalities

Predictor	*b*	95% CI	*t* (*df*)	*p*
(Intercept)	1.69	1.53, 1.84	21.96 (149.00)	<0.001***
time	−0.09	−0.12, −0.06	−5.78 (149.00)	<0.001***
Human video visit	−0.00	−0.22, 0.21	−0.03 (149.00)	0.976
AI* chatbot	−0.12	−0.34, 0.09	−1.12 (149.00)	0.267
AI* avatar	0.00	−0.21, 0.21	0.06 (149.00)	0.955
time * Human video visit	0.01	−0.03, 0.05	0.34 (149.00)	0.713
time * AI* chatbot	−0.03	−0.07, 0.01	−1.38 (149.00)	0.170
time * AI* avatar	−0.04	−0.08, −0.00	−2.01 (149.00)	0.046*

Significance levels: *p < 0.05; **p < 0.01, ***p < 0.001.

A sensitivity analysis was conducted to assess the reliability of the primary outcomes. The model was consistent with the original mixed linear regression model, incorporating random intercepts and slopes for the time variable at the participant level. As in the primary model, a substantial negative effect of time on log-transformed cortisol concentrations (sCort_log_; *β* = -0.091, *p* < .001) was observed. The interaction effect between time and the AI* avatar condition was not statistically significant in the sensitivity model (*β* = -0.035, *p* = .107), but showed a similar direction as in the main model (*β* = -0.042, *p* = .046). On average, male participants showed higher log-transformed sCort concentrations than female participants (*β* = 0.187 *p* = .033). Other covariates, including age, BMI, smoking status, shift work, caffeine consumption, room temperature, and meditation experience, showed no significant effects. The model explained 22.1% of the variance via the fixed effects (pseudo-*R*²_fixed_) and 91.2% of the total variance (pseudo-*R*²_total_), which is very similar to the values in the main model (pseudo-*R*²_fixed_ = 14.6%, pseudo-*R*²_total_ = 90.9%). The intraclass correlation (ICC) value remained high at 0.911 (cf. main model: ICC = 0.917).

**Fig. 2:**
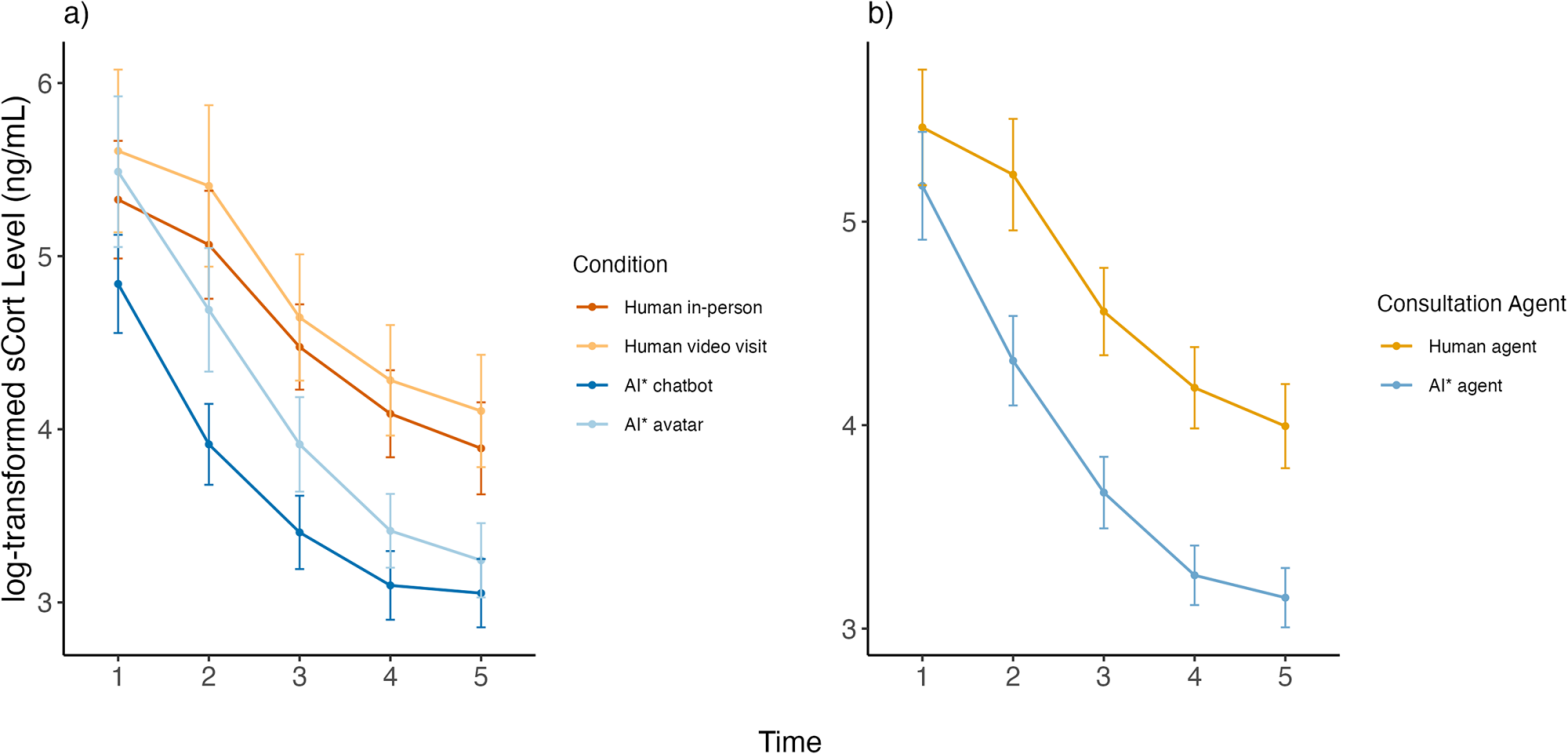
Log-transformed sCort concentrations over time across consultation modalities. **a** shows log-transformed sCort concentrations over time across all four consultation modalities. **b** shows log-transformed sCort concentrations over time across two combined agent conditions. All orange lines represent human physician conditions; all blue lines represent AI physician conditions.

Repeating the analyses combining the four conditions in two agent groups (human vs. AI*) showed the same effects. Log-transformed sCort concentrations decreased over time (*β* = –0.082, *p* < .001). No significant main effect was observed for these two groups. A significant interaction between time and consultation modality was observed for the combined AI* agent group, indicating a stronger decline in sCort over time compared to the human agent group (*β* = –0.040, *p* = .009). The model explained 14.2% of the variance via fixed effects (pseudo-*R*²_fixed_) and 90.8% of the total variance (pseudo-*R*²_total_). In the sensitivity analysis with control variables, all significant effects of the original model remained. In addition, sCort concentrations in males were higher compared to female participants (*β* = 0.194, *p* = .027). This model explained 21.1% (pseudo-*R*²_fixed_) of the variance with fixed effects and 91.1% of the total variance (pseudo-*R*²_total_). Figure 2 shows sCort concentrations over time in the four and two (combined) groups of consultation modality.

### Memory retrieval (H2)

To analyze the effect of consultation modality on memory retrieval in open and closed questions, as well as questions for visual memory, one-way ANOVAs were conducted. Detailed information about memory retrieval is shown in Table 2.

**Table 2:** Memory retrieval across consultation modalities

		Memory Performance		
		open format	closed format	visual memory
Modality	*n*	*M (SD)*	*M (SD)*	*M (SD)*
Human in-person	45	0.43 (0.16)	0.93 (0.11)	0.75 (0.20)
Human video visit	39	0.41 (0.12)	0.96 (0.10)	0.92 (0.16)
AI* chatbot	39	0.39 (0.11)	0.86 (0.15)	0.64 (0.27)
AI* avatar	40	0.43 (0.13)	0.94 (0.11)	0.85 (0.24)
		*F*(3, 153) = 0.83, *p* > 0.05	*F*(3, 153) = 4.71, *p* = 0.004	*F*(3, 83.67) = 12.57, *p* < 0.001

Significance levels: *p < 0.05; **p < 0.01, ***p < 0.001.

Retrieval in open format questions did not differ between groups (*F*(3, 153) = 0.83, *p* > .05, *η²* = .02). Retrieval in closed format questions showed significant differences between groups (*F*(3, 153) = 4.71, *p* = .004, *η²* = .08). Subsequent Tukey post-hoc analyses showed significant differences (adjusted to family-wise error rate) with memory retrieval in closed format questions being significantly lower in the AI* chatbot condition compared to the human video visit condition (diff = −.09, *p* = .003), and to the AI* avatar condition (diff = −.07, *p* = .031). For all other group comparisons, no statistically significant differences were observed (*p* > .05). For visual memory retrieval, a Welch ANOVA was additionally calculated and showed a significant effect of consultation modality on visual memory (*F*(3, 83.67) = 12.57, *p* < .001, *η²* = .31). Post-hoc comparisons using the Games-Howell test showed that the human video visit condition performed significantly better than the human in-person condition (diff = .17, *p* < .001), and the AI* chatbot condition (diff = .28, *p* < .001). Participants in the AI* chatbot condition also performed significantly worse than in the AI* avatar condition (diff = .21, *p* = .003). No other pairwise comparisons were significant.

To test the effect of sCort_AUCi_ on memory scales, three regression analyses were conducted (*H2*). Table 3 summarizes the results of the regression analyses for open format, closed format, and visual memory scores. For open format questions, the interaction between AI* avatar condition and sCort_AUCi_ was significant (*β* = 0.04, *p* = .003), as was the main effect of sCort_AUCi_ (*β* = −0.02, *p* = .026). No significant predictors were found for closed-format questions. For visual memory, the human video visit condition (*β* = 0.22, *p* < .001) and the interaction between the AI* chatbot condition and sCort_AUCi_ (*β* = 0.07, *p* = .003) were significant predictors. The explained variance was highest for the visual memory model (*R*² = 0.28).

**Table 3:** Predictions of memory scales

	Open format	Closed format	Visual memory
Predictor	*ß (p*)	*ß (p*)	*ß (p*)
(Intercept)	0.40 ( < 0.001)	0.93 ( < 0.001)	0.73 ( < 0.001)
Human video visit	−0.00 (0.984)	0.03 (0.432)	0.22 ( < 0.001)***
AI* chatbot	−0.03 (0.450)	−0.04 (0.253)	−0.02 (0.796)
AI* avatar	0.07 (0.051)	0.02 (0.488)	0.11 (0.059)
sCort_AUCi_	−0.02 (0.026) *	−0.00 (0.566)	−0.03 (0.067)
Human video visit*sCort_AUCi_	0.00 (0.893)	0.00 (0.848)	0.03 (0.123)
AI* chatbot*sCort_AUCi_	0.01 (0.611)	0.02 (0.125)	0.07 (0.003)**
AI* avatar*sCort_AUCi_	0.04 (0.003)**	0.01 (0.253)	0.01 (0.492)
*R*^2^	0.10	0.11	0.28
	*F*(7, 140) = 2.16, *p* = 0.041	*F*(7, 140) = 2.51, *p* = 0.018	*F*(7, 140) = 7.77, *p* < 0.001

Significance levels: *p < 0.05; **p < 0.01, ***p < 0.001.

### Perceived credibility of the situation

**Fig. 3:**
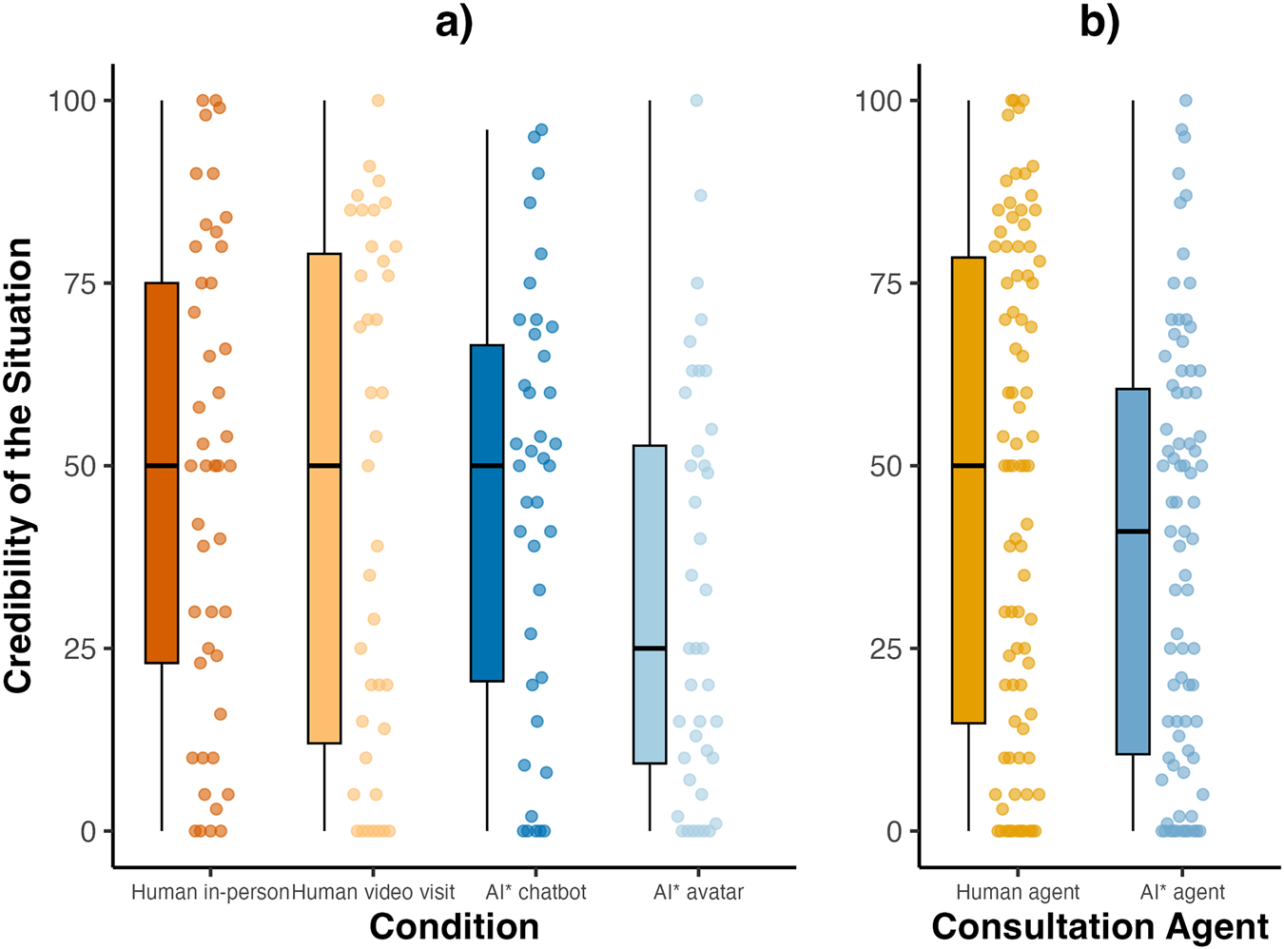
Credibility of the situation across consultation modalities. Panel **a** shows credibility of the situation across all four consultation modalities. Panel **b** shows the credibility of the situation across two combined agent conditions. All orange boxplots and dots represent human physician conditions; all blue boxplots and dots represent AI physician conditions.

A one-way ANOVA was conducted to analyze the effect of all four conditions on perceived credibility of the situation. The analysis revealed a non-significant difference in perceived credibility of the situation between the four conditions (*F*(3, 159) = 2.18, *p* = .093, *η²* = .04). Subsequent Tukey post hoc analyses showed no significant differences between human in-person (*M* = 47.89, SD = 32.29), human video visit (*M* = 45.33, SD = 34.90), AI* chatbot (*M* = 44.95, SD = 28.99), and AI* avatar settings (*M* = 31.90, SD = 27.91). Again, this analysis was repeated with two combined agent groups (human vs. AI*). In the human agent group, normality was not met (*W* = .92, *p* < .001). A Wilcoxon Rank test showed no significant difference between the human agent (*M* = 46.70, SD = 33.35) and AI* agent group (*M* = 38.34, SD = 29.02, *W* = 3816, *p* = .098, *d* = -.27). Even though it is not statistically significant, participants in the AI* agent group seem to report lower perceived credibility of the situation compared to participants in the human agent group. Figure 3 shows the credibility of the situation across conditions. Panel a presents all four conditions, whereas panel b shows the two combined agent groups.

Two multiple linear regressions were conducted to analyze the effects of all four conditions, perceived credibility of the situation, and their interactions on subjective stress and sCort_AUCI_. For predicting subjective stress, the overall model was significant (*F*(7, 155) = 8.29, *p* < .001) with *R*^*2*^ = .27. Compared to the reference group in-person, no other condition significantly affects subjective stress (human video visit: *β* = 8.27, *SE* = 8.34, *p* = .323; AI* chatbot: *β* = −2.52, *SE* = 8.87, *p* = .777; AI* avatar: *β* = 9.57, SE = 7.99, *p* = .233). Credibility of the situation showed a significant positive main effect on subjective stress (*β* = 0.52, SE = 0.10, *p* < .001) and a significant interaction with AI* avatar condition (*β* = −0.36, SE = 0.16, *p* = .027), resulting in higher subjective stress levels with low credibility of the situation for participants in the AI* avatar condition. All other interactions between credibility of the situation and condition on subjective stress were not significant (credibility * human video visit: *β* = -0.14, SE = 0.15, *p* = .323; credibility * AI* chatbot: *β* = −0.18, SE = 0.16, *p* = .259).

**Fig. 4:**
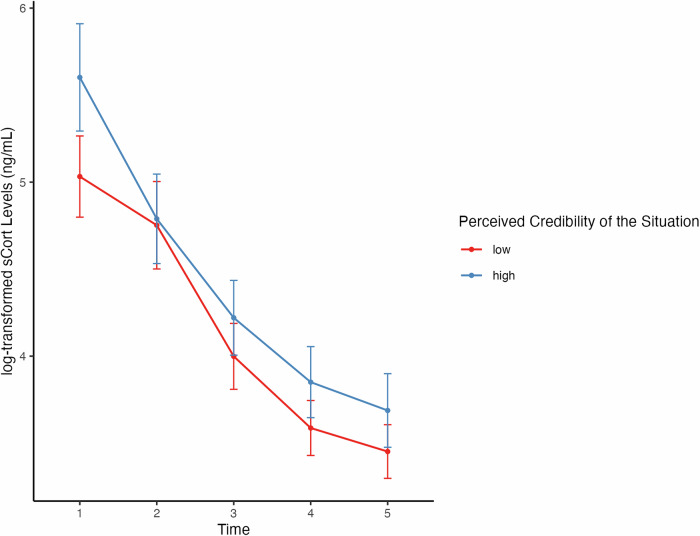
Log-transformed sCort concentrations by perceived credibility of the situation. Log-transformed sCort concentrations over time, split by median of low (red line) and high (blue line) perceived credibility of the situation. Bars indicate standard errors.

For predicting sCort_AUCi_, the overall model was not significant (*F*(7, 141) = 1.74, *p* = .104), with *R*² = .08, indicating that only 8% of the variance in sCort_AUCi_ was explained by the predictors. None of the predictors reached statistical significance. Specifically, compared to the reference group (human in-person), the human video visit (*β* = −0.44, *SE* = 0.90, *p* = .621), AI* chatbot (*β* = -1.57, SE = 0.97, *p* = .105), and AI* avatar condition (*β* = -0.63, SE = 0.87, *p* = .474) were not significantly associated with sCort_AUCi_. The main effect of credibility of the situation (*β* = -0.02, SE = 0.01, *p* = .112) and the interaction terms (credibility * human video visit: *β* = 0.01, *SE* = 0.02, *p* = .401; credibility * AI* chatbot: *β* = 0.02, SE = 0.02, *p* = .234; credibility * AI* avatar: *β* = −0.01, *SE* = 0.02, *p* = .552) were also not significant. Figure 4 shows the sCort curves separately for high and low credibility (median split), indicating a trend of perceived credibility of the situation on a descriptive level.

### Exploratory analyses: sex differences

Subjective stress, sCort_AUCi_, credibility of the situation and memory scales were tested for sex differences. No comparison revealed significant effects (see Table 4).

**Table 4:** Sex differences in main variables

Variable	Sex	*M* (SD)	Test	Statistics	*p*	Cohen’s *d*
Subjective stress	Female	57.04 (24.87)	Wilcoxon	*W* = 1992.50	0.318	−0.17
	Male	61.27 (27.12)				
sCort_AUCi_	Female	−1.58 (2.18)	Wilcoxon	*W* = 1773	0.521	−0.12
	Male	−1.30 (2.62)				
Perceived credibility of the situation	Female	44.63 (31.70)	Welch t-Test	*t*(56.31) = 1.58	0.119	0.29
	Male	35.43 (30.13)				
Memory Open Format	Female	0.42 (0.13)	Welch t-Test	*t*(51.95) = 1.33	0.188	0.26
	Male	0.39 (0.14)				
Memory Closed Format	Female	0.92 (0.13)	Welch *t*-Test	*t*(64.20) = −0.98	0.332	−0.17
	Male	0.94 (0.10)				
Visual Memory	Female	0.78 (0.25)	Welch *t*-Test	*t*(58.06) = −1.49	0.142	−0.27
	Male	0.84 (0.22)				

Gender differences were tested for *n*_Female_ = 128 and *n*_Male_ = 35. Wilcoxon rank test and Welch *t*-test were used depending on the distribution and homogeneity of variances.

## Discussion

This study investigated the impact of different conversation modalities in simulated medical consultations—ranging from human in-person and video-based human interactions to AI-styled chatbot and avatar formats—on subjective stress, salivary cortisol, memory retrieval, and perceived credibility of the situation. The present study contributes to this discussion by examining responses to specific, simulated interaction configurations within a controlled experimental setting, focusing on participants’ perceptions and expectations associated with interacting with an entity believed to be AI. Overall and taken together, the results indicate that the different consultation modalities affected stress, memory, and credibility in partly distinct ways. Human-physician consultations elicited higher subjective stress than AI*-physician consultations, while salivary cortisol levels declined over time in all conditions, with a stronger decrease in the AI*-physician formats. Memory performance did not follow the same pattern: open-format retrieval did not differ between consultation modalities, memory in closed-format questions was lower in the AI* chatbot condition, and visual memory was highest in the human video visit. Perceived credibility was comparable across the human in-person, human video visit, and AI* chatbot conditions but lower in the AI* avatar condition. Higher credibility was associated with higher subjective stress; only participants of the AI* avatar condition showed reversed effects. Overall, the results suggest that lower stress in AI*-based consultations does not necessarily translate into better memory performance, and that stress–memory relations may depend on the specific consultation modality rather than showing a uniform pattern across conditions.

For subjective stress, a main effect of consultation modality was found, indicating that participants reported higher stress levels in interactions with human physicians compared to the AI* physician conditions within the simulated consultations. This finding contradicts our initial hypothesis (H1), which predicted higher stress in AI*-physician conditions due to assumptions regarding the impersonal nature of AI-mediated interactions. Similarly, salivary cortisol concentrations declined more rapidly in AI*-physician conditions compared to human-physician conditions, but differences in the overall decline were not observed. According to the social self-preservation system^45^, the prospect of genetic counseling may have induced a sense of uncontrollability and social-evaluative threat. However, since baseline values were identical across groups, the significant interaction of time and condition confirms that the subsequent cortisol declines were not a mere passive wash-out, but were actively moderated by the interaction modality. While future research should include person-specific baselines outside the laboratory to better isolate these anticipatory effects from true physiological rest^46^, the present data suggest that the interaction itself drove the physiological change. This finding is inconsistent with hypothesis H1, which assumed that consultations with an AI*-physician would lead to greater physiological arousal. However, the literature on stress in medical communication indicates another direction. Studies on virtual embodiment suggest that realistic avatars may increase emotional arousal^28^, and research on remote consultations with a human physician via telephone or video call can actually reduce subjective stress^26,47^. In addition, insensitive communication of bad news that might be more likely in AI-based conversations can lead to higher stress levels in patients^5^ and the way of communicating with patients has the potential to impact patients’ autonomic arousal^48^. Although previous studies suggest that the delivery of bad news through AI systems might lead to increased stress in patients, the present study did not find higher stress levels in the simulated AI*-physician modalities. In contrast, a recent study showed that chatbot responses were perceived as more empathic than responses of actual physicians in a sample of cancer patients^49^. While AI agents can simulate empathy convincingly, this empathy remains programmed rather than experienced. Perceived empathic communication may therefore reflect simulation rather than genuine emotional understanding^50^. Previous research showed that medical consultations on intimate topics were preferred to be digital instead of personal^24,51^. Digital or AI-based consultations could convey a sense of anonymity and create a space without judgment or social pressure. Prior research suggests that individuals tend to disclose more when interacting with AI agents that are perceived as nonjudgmental^52^. Within the context of the simulated consultations examined in this study, such mechanisms could help explain the reduced stress levels in AI*-physician conditions in this study. Furthermore, the higher subjective stress in the human conditions might reflect subtle social discomfort within the experimental setup. Although participants did not actively role-play, interacting with a real human inherently introduces elements of social evaluation and performance anxiety that may be reduced with an AI agent. Future studies should therefore distinguish between task-induced and social evaluative stress in automated versus human consultations.

The present study found no significant main effects of conversation modality on open-ended memory retrieval, although higher cortisol concentrations tended to impair memory retrieval for open-format questions overall, which is in line with our second hypothesis (H2). Only in the AI* avatar condition, participants with higher cortisol concentrations tended to recall more. For multiple-choice questions (recognition memory), no effect of conversation modality or cortisol concentrations was found. Visual memory showed the biggest differences in this study, with the highest visual memory retrieval for participants in human video visits. We even found an interaction between cortisol concentrations and AI* chatbot group, resulting in higher visual memory for more stressed participants when communicating with an AI* chatbot. Studies on human-to-human medical conversations showed that supportive and affective communication^40,53^ as well as trust-conveying communication^38^ improves free memory recall and recognition^53^. These results suggest that effects in patients’ memory might depend more on the quality of the interaction than the type of conversation modality. The present study used standardized medical consultations that did not vary in the communication style, which allows for comparisons to other studies on patient memory in telemedical settings. Another study on giving instructions via telehealth versus in-person conversations showed poorer retrieval in virtual formats^54^, which contradicts the present study’s results on visual memory retrieval. Overall, interacting with an AI* chatbot led to lower memory retrieval. Following the Dual Coding Theory^55^, information that is presented verbally and visually should lead to a better recall. In our case, participants in the AI* chatbot received information as written text that is visually presented but verbally encoded. In addition, the information was threatening to one’s health status and therefore emotionally relevant. Due to the emotional strain during the conversation, working memory may have been impaired, potentially contributing to difficulties in retrieving the discussed content^56^. This would be in line with the Emotional Impairment Hypothesis: Emotional content impairs the ability to maintain information in working memory, likely because it consumes cognitive resources and diverts attention away from the primary task^57^. Furthermore, the divergent findings between visual and verbal memory scales might reflect a trade-off in the allocation of limited cognitive resources. It is possible that in visually rich environments, such as the human video visit, attention is partially directed toward processing environmental cues, potentially at the expense of encoding the specific verbal details of the medical consultation. This distinction between environment-focused and content-focused memory processing warrants further investigation in digital health communications. Other research showed that the timing of stress is relevant for consolidation processes. Especially for episodic memory, stress shortly before, during, or after encoding leads to better consolidation of memory via cortisol and norepinephrine^42^. This study’s idea of using simulated AI-styled agents as mediators of knowledge is linked to the potential of AI applications to better inform patients, thereby enabling them to act more autonomously and supporting shared decision-making. However, research remains divided on this issue. Some studies argue that AI applications can enhance shared decision-making by promoting patient autonomy, while others caution that it may suppress value plurality and inadvertently reinforce paternalism^58^. Studies on AI-enabled decision aids have shown increased patient engagement and a stronger sense of control, yet clinicians have raised concerns about the provision of outdated information and the risk of inadequate treatment recommendations^59^. Building on these debates, As’ad^60^ proposes a conceptual framework in which AI functions as a knowledge facilitator between clinicians and patients, aiming to sustain and facilitate shared decision-making rather than replace it. Existing research largely conceptualizes AI systems as knowledge bases or information facilitators rather than as full conversational partners. Future studies should examine whether AI physicians could support patients who prefer the informative model of care by providing consistent and unlimited access to explanations, a question that remains empirically open. Further research on memory performance in analog versus digital settings, as well as across different digital and AI-based modalities, is needed.

Hypothesis 2 proposed that cortisol concentrations would moderate the relationship between conversation modality and memory retrieval. This assumption was partially supported. The present study found that higher cortisol tended to reduce open-format retrieval overall. However, participants in the AI* avatar group with greater cortisol concentrations retrieved more information. For visual memory, participants in the AI* chatbot condition retrieved more information with stronger cortisol responses. The overall effect of higher cortisol concentrations impairing retrieval is in line with previous research^37,43^. These findings suggest that the relationship between cortisol and memory retrieval may be influenced by the specific consultation modality. Within this simulation, configurations presented as AI* were associated with lower subjective stress and a more rapid decline in cortisol compared to human conditions. However, as memory outcomes were mixed, these interactions should be interpreted cautiously. The relationship between physiological arousal and information processing may be moderated by consultation modality. Specifically, the interplay of reduced social complexity and personal health relevance—reflected in the slower cortisol decline under high perceived credibility—could play a decisive role: both constructs were significantly less pronounced in the AI* physician settings. While this might tentatively align with concepts like attentional narrowing^44^, further research is needed to determine how these specific configurations consistently impact the encoding and retrieval of medical information under stress.

In the present study, human in-person consultations, human video visits, and AI* chatbot consultations were rated similarly in perceived credibility of the situation. The AI* avatar condition received the lowest credibility ratings. This aligns with evidence that individuals prefer and trust a human physician more than AI-physicians^24,25,61^. As the AI* chatbot condition received comparable ratings on credibility to human-physician conditions, this effect may have been driven by the representation of the agent (virtual avatar) and not the agent type itself. Research shows that anthropomorphic features of virtual avatars are relevant to how they are perceived^62^. However, in the present study, the interaction with an AI* avatar lacked sensory or social cues, which might be the reason for its lower ratings in perceived credibility of the situation. Research on virtual embodiment shows that a high sense of embodiment in virtual reality can amplify affective arousal to virtual stimuli^28^. When a simulated medical consultation feels more embodied, patients may perceive it as a real social interaction. As a result, they might be more likely to appraise the situation as credible and emotionally relevant. In the present study, higher ratings of perceived credibility were associated with higher levels of subjective stress and showed a tendency toward higher cortisol concentrations. These findings suggest that greater perceived realism leads to deeper emotional engagement with the consultation content. Studies examining the delivery of screening results in clinical practice indicate that patients generally prefer receiving results through direct, personal interaction with their physician^63,64^. For non-critical results, telephone communication is considered an acceptable alternative^63^. The use of written communication, such as letters, is becoming more common; however, this method can make it more difficult for patients to ask questions and may increase uncertainty and emotional burden. Especially in emotionally sensitive situations, it is important to keep the patient at the center of care and to ensure that sufficient support is provided.

In the present study, the WOZ approach was not intended to provide a validated simulation of existing AI systems or their technical capabilities. Instead, its purpose was to examine how participants respond under the assumption of interacting with an AI physician, focusing on subjective interpretation rather than objective system fidelity. Information from the debriefing suggested that some participants expressed uncertainty about the nature of the interaction partner, often in terms of perceived pauses or atypical conversational behavior, rather than explicitly attributing control to a human operator. Most participants did not report actively questioning the system’s autonomy during the interaction, indicating that they generally engaged with the system under the intended experimental framing, despite individual variation in perceived realism. Consistent with this pattern, perceived realism differed across conditions, with the avatar-based interaction being rated as less realistic than all other modalities (see Supplementary Materials E)). Accordingly, the findings should be interpreted as reflecting responses to this specific, simulated AI*-physician setup, situated within a defined technological context, rather than as evidence about AI systems as deployed in clinical practice. Furthermore, it is important to note that the study design compares four distinct consultation configurations rather than isolated variables. Consequently, it is not possible to disentangle whether the observed effects stem from the agent type itself or from differences in communication modality, social presence, and embodiment. Given that the study was conducted prior to the widespread availability of advanced dialog-based AI systems, external validity with respect to these systems may be limited. Future research may build on this work by systematically varying degrees of autonomy, realism, and standardization across AI-based interaction paradigms to assess how such design choices shape patient experience. From the perspective of generalizability, the findings are particularly informative because the WOZ design enables a robust empirical evaluation before technical limitations (e.g., error-prone speech recognition or incomplete dialog models) distort interaction quality and, consequently, the interpretable impact of the system. This allows the study to capture the intended functionality of the avatar concept under realistic interaction conditions and supports a valid estimation of its practical potential in everyday clinical practice. By methodologically decoupling technological maturity from the user experience, external validity is strengthened insofar as the observed effects can be attributed primarily to the interaction and communication design—i.e., to those components that will also be pivotal in the eventual deployment context.

Several important methodological limitations of this study should be explicitly acknowledged. Both AI* modalities in this study were implemented using a WOZ approach, with human operators generating real-time responses. This methodological choice represents a limitation, as the simulated agents do not capture the full range of constraints and behaviors of real-world AI systems. Accordingly, several potentially relevant dimensions of variation on the intervention side (e.g., linguistic style, physician’s gender, or interface-related features) were deliberately held constant and not examined independently in this study. At the same time, the study design enabled responses that were appropriate to the conversational context, while ensuring a controlled and consistent tone and content across all experimental conditions. In this context, participant sampling and statistical analysis were intended to support robust inference about participants’ responses to the configurations of this study, rather than to imply generalizability of the simulated AI systems. Importantly, the WOZ setup was not intended to provide a validated approximation of AI systems, but rather to enable a controlled examination of participants’ responses to specific, simulated interaction configurations while minimizing variability stemming from technical system behaviors not under investigation in this study. Consequently, the findings should be interpreted as reflecting the psychological perspective, i.e., the individual interpretation while interacting with an AI, rather than AI systems as constantly adapted and deployed in clinical practice. Since the time of data collection, the technological landscape of AI communication systems has advanced considerably. Recent progress in realistic voice synthesis and expressive virtual avatars (e.g., Resemble AI for voice cloning or HeyGen for realistic virtual avatars) has improved the realism and interactivity of AI representations. Additionally, with the development of large language models (LLMs), AI systems can support increasingly complex, dialog-based interactions and clinical decision making^65^. It can be assumed that due to the fast uptake and widespread availability of LLMs at the time of writing, the general population today feels more positive towards interacting with AI systems than they did at the time of the experiment. In light of these developments, future research might build on these findings by examining a wider range of AI-based interaction approaches. Furthermore, future studies should explicitly assess participants’ prior experience with AI and their trust in automated healthcare tools. As the current study was conducted in 2022, participants likely had minimal exposure to advanced AI systems, which may have resulted in different expectations and stress responses compared to today’s populations. Specifically, during the time of data collection prior to the widespread public familiarity with Large Language Models, participants’ baseline expectations regarding an AI’s clinical accuracy, social understanding, or capacity for cooperation were likely lower or more abstract. Modern LLM-based systems may intentionally allow for less experimental control in order to achieve greater ecological validity. Such approaches may be especially useful for understanding how patients actually experience emerging AI systems in real-world contexts, even when it is then not possible to fully standardize conversational content.

Recognizing that a scripted simulation may not fully capture the complexities inherent in real-world physician-patient interactions, this study measured the participants’ subjective credibility of the simulated situation. While differences were not statistically significant, the lower credibility level of the AI* avatar condition—with all other three conditions showing very similar credibility levels—could be seen as an indication that the simulation of this condition was less successful, potentially limiting the generalizability of the results for other avatars beyond the WOZ avatar in this study. Furthermore, given that there was a very wide variance in situational credibility amongst all four conditions, it can be assumed that the subjective experience of the simulated situation differed widely amongst the participants, independently of the consultation modality. Additionally, the average credibility scores across all conditions were below the scale midpoint. This suggests that participants did not experience the simulated consultations as fully equivalent to real clinical encounters. Consequently, it remains uncertain whether the observed stress and memory responses would generalize to interactions with actual physicians or real-world AI systems. Future studies should therefore examine these effects in settings with higher ecological validity.

A further limitation concerns visual differences across the physician representations used in the study. The consultation modalities differed not only in interaction format but also in size of the interaction partner, framing, and apparent interpersonal distance. This may have influenced the participants’ impressions independently of the content of the conversation. Moreover, variations in the physical appearance of the researchers portraying the physicians may have introduced further uncontrolled variability. Future studies should more tightly standardize visual features such as face size, framing, and viewing distance across conditions.

The sample of the present study consisted primarily of young women. While this gender balance might well present the gender ratio in medical consultations, younger individuals tend to be more familiar and comfortable with digital technologies, and their experiences with AI-based consultations may not generalize to older populations. Future research should include participants with a broader age range and ensure a more balanced gender distribution to ensure the results reflect real-world diversity in patient attitudes and stress responses. Since all participants were healthy and aware that they were not undergoing a real medical examination, the observed responses may not fully reflect those in actual clinical contexts, particularly during the delivery of serious news. This reliance on a healthy sample was necessary to ensure valid salivary cortisol measurements, yet it limits the representativeness of the findings. Furthermore, the use of Wizard of Oz simulations for the AI* conditions means the results may not directly translate to the routine use of fully autonomous AI systems. However, given the early stage of research in this field, this simulated approach represents an ethically appropriate first step. It allows for a controlled investigation of sensitive interactions without exposing patients to unvalidated communication methods. While this study focused only on the delivery of bad medical news, future research could also investigate medical consultations that are less emotionally burdensome. Also, participants’ prior familiarity with Huntington’s disease was not formally assessed, which may have influenced individual emotional reactivity to the news. Future studies should screen for such prior knowledge to better control for variations in stress responses.

In line with open science principles, this study was pre-registered to prevent exploratory multi-testing. Because we investigated distinct psychological and physiological constructs, a study-wide alpha-correction was not applied to preserve statistical power; however, we strongly encourage future independent replication studies to further establish the robustness of these individual effects.

Despite its limitations, the study has notable strengths. A recent review on in-person versus online video consultations for surgery found no study investigating information retrieval in patients^66^. Our study provides first insights on this topic, as technical possibilities develop at a fast pace and provide easy access to telemedical services.

The study revealed meaningful differences between simulated conversation modalities, with healthy participants perceiving the situation as sufficiently credible to support the validity of simulation-based research in emotionally charged medical scenarios. This suggests that simulated interactions can be a useful research tool, especially in cases where studying real interactions would not be ethically appropriate.

The present study shows that, within the simulated consultations examined, interacting with an AI* physician in an emotionally challenging situation caused less stress than interacting with a human physician. These findings could help inform discussions around the design of conversational technologies intended to support patients who experience anxiety during medical appointments. Rather than replacing human physicians, conversational AI systems could complement traditional care – for instance, by providing a low-threshold follow-up option after consultations. This may be particularly beneficial for patients who are hesitant to re-contact their human physician, and could also help overcome barriers such as limited availability or access. In addition, future studies should examine less emotionally demanding contexts to allow for more precise conclusions about the potential use cases of conversational technologies in routine care.

Our findings indicate that different formats of medical consultation may be associated with varying levels of subjective and biological stress responses and modality-specific patterns in memory retrieval. These results suggest that conversational technologies should not be evaluated solely in terms of technical feasibility or user acceptance, but also with regard to their psychological effects on patients. As healthcare increasingly integrates digital communication tools, it will be essential to understand how interaction modalities relate to emotional burden, cognitive processing, and the effective delivery of sensitive medical information.

## Methods

### Study design

The data in this study were acquired within a larger interventional trial (pre-registered at OSF^67^). Data collection took place at the Core Facility for Neurocognitive Systems Research ((CNSR), Heidelberg University) from May to October 2022. The study was conducted in accordance with the Declaration of Helsinki and was approved by the Ethics Committee of the Medical Faculty Heidelberg (Ethics approval number: S-067/2022). All participants provided written informed consent before participation. Details on participant safety, ethical management, and debriefing procedures are provided in the Supplementary Information A).

### Experimental procedure and simulation approach

This study employed a standardized protocol simulating the communication of genetic test results for Huntington’s disease to ensure identical conditions across consultation modalities. Therefore, all participants received the same information. The detailed script of the simulated consultation is available in the Supplementary Materials. Huntington’s disease was chosen because it allows for a realistic diagnostic simulation in healthy volunteers, as the condition can be detected decades before the onset of physical symptoms^68^. This rare neurodegenerative disorder provided a severe yet credible scenario for asymptomatic participants.

Each participant took part in two separate interviews. Throughout both sessions, saliva samples were collected. Both interviews followed a pre-standardized structure to ensure consistency and to enable memory-retrieval assessment. In the first interview, participants were informed, as part of the role-play, about a new screening procedure for Huntington’s disease and were told that their initial saliva sample indicated a 1/3 probability of having the disease. This feedback was fictitious; no saliva samples were analyzed at this stage. After a 5–10 min break, the second interview communicated a positive genetic test result, described as having been performed on-site using their saliva sample. No real genetic testing took place in this study. Both interviews covered the nature of Huntington’s disease and its symptoms, with identical content across participants addressing particularly the cytosine-adenine-guanine CAG trinucleotide repeat, dependent age at onset, progression, treatment options, and potential life impacts. The only customization occurred during the pedigree analysis, which explored possible familial evidence of the disease and aimed to enhance participants’ immersion in the simulated consultation.

Before the interviews, participants were informed about the study’s purpose and provided written consent. This included information stating that they would not undergo a real medical examination or any genetic testing. After the experiment, they were debriefed to again underline the nature and purpose of the roleplay, allowing them to ask questions. Saliva samples were collected at five time points: before the first interview, between interviews, after the second interview, following completion of the questionnaire, and immediately prior to departure. All saliva samples were stored at −80 °C for later analysis. Participants completed the online questionnaires on a provided laptop using the Unipark survey platform (Tivian XI GmbH, Frankfurt am Main, Germany). Throughout all interviews, the physician wore a white coat to maintain a consistent professional appearance. To preserve anonymity while ensuring correct attribution of test results, participants were not addressed by name but were identified only by their subject code. Upon study completion, participants received a monetary compensation of 25 Euros.

### Consultation conditions and implementation of the Wizard of Oz (WOZ)

This study combined standardized interviews with saliva sampling to enable a controlled investigation of participants’ responses to genetic test results across different consultation modalities. The simulated genetic counseling consultations were conducted in conditions displaying four modalities: in person with a human physician, via video visit with a human physician, via text-based chatbot with an AI* physician, or via spoken virtual avatar with an AI* physician. The physicians in this study were impersonated by one of three researchers of the study team (TLS, TRK, TIS). Participants were randomly assigned to one interaction condition and were blind to the other conditions. Due to the nature of the experimental design, blinding of the study team was not feasible, as team members needed to be aware of the assigned condition to conduct the experiment. Table 5 provides a short overview of conditions displaying different consultation modalities.

**Table 5:** Overview of consultation modalities

Condition	Agent	Modality	Representation	Implementation
Human In-person	Human	Spoken (in-person)	Physical human body	In-person interaction with a human physician
Human Video Visit	Human	Spoken (audio-visual)	Real human visible in real-time video	Video call via secure Webex connection
AI* Chatbot	AI	Text-based	Screenshot of the avatar as a profile picture	Authors NM and JM developed the participant and operator interfaces^42^. Participants could read messages and type their responses. The operator could select from pre-written script segments (see Supplementary Materials A), type additional responses as needed or share images.
AI* Avatar	AI	Spoken through a simulated voice (audio-visual)	A virtual humanlike figure on the screen with interactive facial expressions and simple gestures	Video call via a secure Webex video connection; however, the operator’s video was not shared. Instead, the operator shared the screen displaying the avatar of the AI physician (Virtual Human Toolkit^43^). One of the authors (DG) developed an operator interface, analogous to that used in the chatbot condition, which allowed the operator to select from pre-written script segments (see Supplementary Material A) or type additional responses.

In the in-person condition, the participant and physician sat across from each other at a table in a designated room. The physician accessed test results on a clipboard, and the pedigree was recorded manually during the session. In the video visit condition, with a human physician, two separate rooms were used: the participant was alone with a laptop, while the physician was in another room. Participants were told that the consultation would take place virtually, using a conference call. It was not clarified where the physician was based, but it was ensured that participants would not see the physician beforehand to keep up the impression of a remote consultation. Communication occurred over a secure Webex connection (Cisco Systems Inc.), and the pedigree was shared via screen sharing.

The two conditions interacting with an AI physician simulated future technology-mediated consultations using a WOZ design. Throughout this paper, the notation AI* is used to distinguish simulated AI conditions from interactions with actual AI systems. Participants were told that the consultation they were about to have was going to be led by an autonomous AI* physician (the conversational agent), while a trained operator (wizard) controlled the interaction to ensure consistent quality and standardization. WOZ designs are already used to test telemedical settings. They have been applied, for example, to simulate the automatic collection and preparation of patient information^69^, to address patients’ mental health topics^70^, to support medication adherence^71^, and to provide robotic assistance for individuals with physical disabilities^72^. The simulated AI* conditions in this study were implemented as fixed interaction configurations and treated as experimental stimuli, rather than as representations of a population of AI systems. For the manipulation check, we assessed the perceived credibility of the situation. Later, in the debriefing, participants were informed about the real interaction partners “behind” the assumed AI* physician and asked about their personal impressions. For the AI* chatbot condition, a custom secure web interface was developed by two of the authors (NM and JM). Participants and the wizard were in separate rooms. The participants’ interface supported viewing and replying by typing to the AI* physician’s text messages. The wizard’s interface included pre-scripted conversation elements and the ability to input custom responses if needed. For the avatar condition, an adapted version of the Virtual Human Toolkit^73^ (VHT) was used. An application with embedded text modules, similar to the AI* chatbot system, was developed for the wizard, who used a dual-screen setup to control the avatar’s spoken responses and monitor output. Participants viewed the virtual avatar on their screen, with communication facilitated via secure Webex. The conversation between the participant and the AI* avatar was conducted exclusively via verbal communication and did not include a text-based chat. The pedigree was displayed on the screen using the same graphical style as in all other experimental conditions to ensure visual consistency. The wizard could observe participants’ reactions through the video feed. Screenshots of AI* chatbot and AI* avatar interfaces are provided in Supplementary Materials C). Several measures were taken to calibrate the WOZ design. First, the WOZ system used a clear dialog structure and predefined (canned) responses in the wizard’s user interface^74,75^. To create a convincing simulation, the wizard was visually and acoustically anonymized: in the AI* avatar condition via the VHT, and in the AI* chatbot condition via the chatbot interface, thereby technically masking the wizard^75^. All three wizards completed a standardized wizard training to ensure consistent simulations^see^^76^. The standardized wizard training included several simulated practice runs to calibrate response times and ensure that the predefined dialog structure covered all potential participant inquiries effectively. In addition, the wizards’ perception of the participant was restricted: they could only rely on typed responses in the AI* chatbot condition and on camera and microphone input in the AI* avatar condition^76^. This restriction was implemented to support external validity by limiting the wizard’s access to sensor inputs available to real AI systems^see^^76^.

For the human-physician conditions, the role of the physician was impersonated by one of three medical researchers (TLS, TRK, TIS), all male and of a similar age. The same three researchers also acted as wizards in the AI*-physician conditions. The three researchers were randomly assigned to the experimental conditions, with assignments balanced across conditions. Several measures were taken to standardize both the flow of each consultation and the surrounding procedure. Each consultation, both for human and for AI*-physician conditions, followed a standardized script that the researchers impersonating the physicians and wizards had practiced multiple times before, ensuring that items were standardized for high internal validity (see Supplementary Material B). For internal validation, a part of the study team that did not act as wizards or physicians in the experiment reviewed the consultations afterward for completeness of the reported content and adherence to the predefined script. Any meaningful deviation from the script led to exclusion of the corresponding items from analysis. If less than 80% of the information specified in the script was conveyed to participants, those individuals were subsequently excluded from the analysis. Also, human and AI* avatar physicians had a similar appearance, which included a neutral physician’s white coat or a white shirt, long trousers, no stethoscope, brown hair, and male phenotype. In the AI* chatbot condition, the chat displayed a static image of the avatar used in the AI* avatar condition, to provide participants with a similar visual impression. Likewise, the entire study team was briefed to strictly adhere to a study protocol, which had predefined steps in the same manner for every participant. Any meaningful deviation from the standardized procedure led to exclusion of the corresponding item from analysis.

### Participant recruitment and inclusion criteria

Participants were recruited through various sources in Heidelberg, Germany, including public institutions, private residences, and through the websites of Heidelberg University Hospital and the Institute of Medical Psychology (Heidelberg University Hospital). Flyers were distributed, and online advertisements were used to reach potential participants.

Eligibility was assessed in a short telephone interview conducted several days before the laboratory appointment. During this interview, candidates completed a brief self-report checklist that collected information on age (in years), sex (0 = male, 1 = female), BMI (kg/m²), smoking status (no/yes), and intake of hormonal contraceptives (no / yes). Candidates who did not meet these criteria were not invited to participate in the experimental session.

**Fig. 5:**
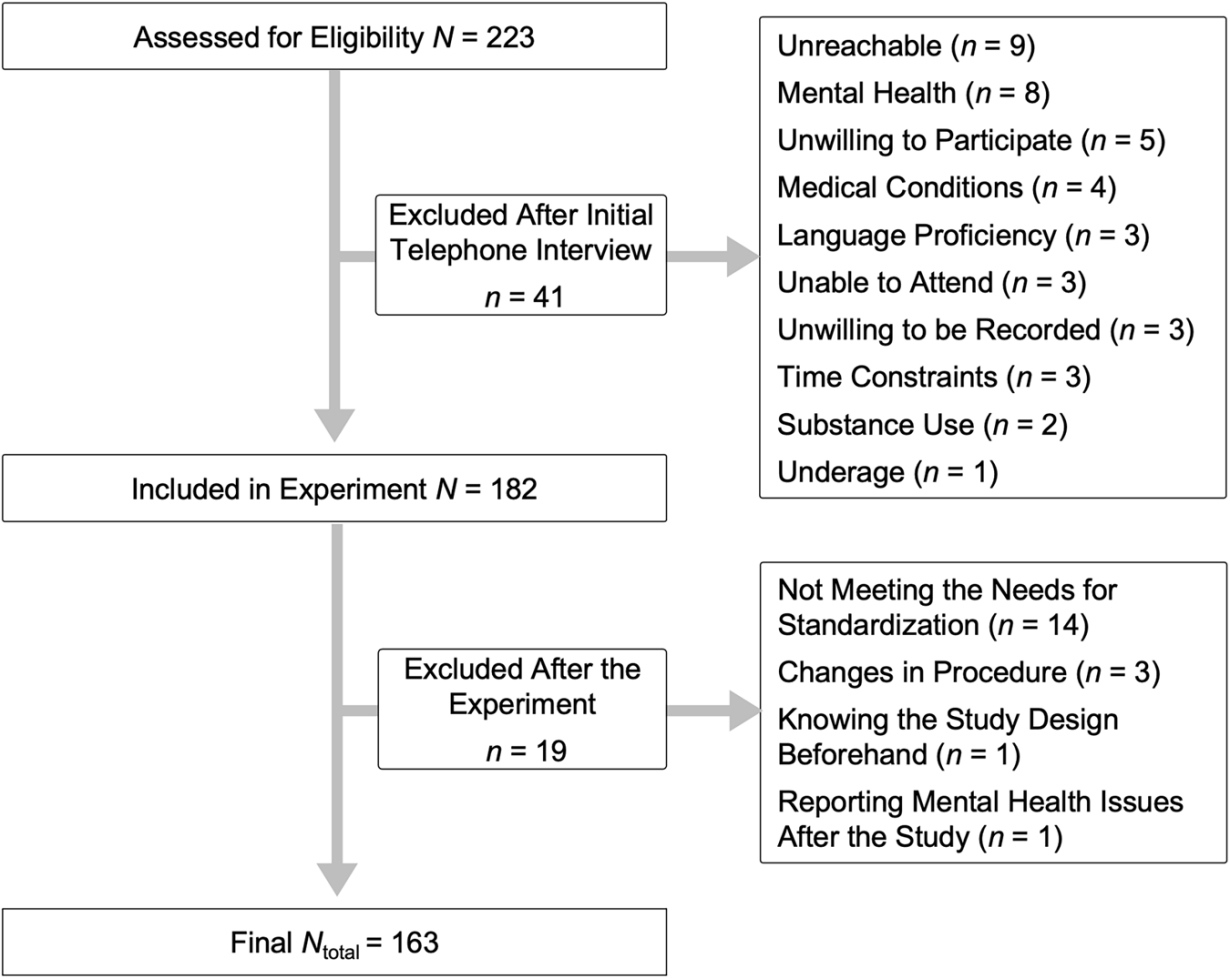
Participant flowchart. This figure illustrates the full participant flow from recruitment to final inclusion in the analysis.

Participants were excluded if they reported chronic severe somatic diseases (e.g., cancer), chronic and severe psychiatric disorders, heavy smoking (≥20 cigarettes per day), intake of psychotropic medication, excessive caffeine consumption (more than 8 cups of coffee or caffeinated beverages per day), severe sleep disturbance within the prior month, autoimmune or major health disorders, current sickness or sickness over the past week, or pregnancy. Additionally, participants were required to be of legal age, able to provide informed consent, and have good knowledge of written and spoken German. To be included, participants were instructed to avoid eating or drinking anything besides water and to refrain from exercise within 2 h before the study. Participant enrollment and exclusion are summarized in the flow diagram (Fig. 5).

### Sample size and characteristics

To determine the required sample size, an a priori power analysis was conducted using G*Power^77^. Based on subjective stress and the area under the curve (AUC) of salivary cortisol as the primary outcomes, a one-way ANOVA with four groups was assumed. With a medium effect size of *f* = 0.30, a power of 0.80, and an alpha level of 0.05, the analysis yielded a required sample size of 128 participants. To allow for possible data loss—whether due to participant dropout, technical difficulties, or issues related to protocol adherence or eligibility on specific testing days—a larger sample of *N* = 150 was planned. The size of the participant sample was intended to support robust inference about participants’ responses to the specific interaction configurations examined in this study, rather than to imply generalizability of the simulated AI systems themselves. Table 6 provides an overview of sample characteristics.

**Table 6:** Sample characteristics

	Human in-person	Human video visit	AI* chatbot	AI* avatar	Overall
	(*n* = 45)	(*n* = 39)	(*n* = 39)	(*n* = 40)	(*N* = 163)
Age					
*M* (SD)	26.3 (9.91)	24.7 (9.29)	24.9 (7.84)	27.4 (9.74)	25.9 (9.24)
Median [min, max]	23.0 [19.0, 61.0]	22.0 [18.0, 66.0]	23.0 [18.0, 58.0]	23.0 [19.0, 54.0]	23.0 [18.0, 66.0]
Sex					
Female	36 (80.0%)	31 (79.5%)	30 (76.9%)	31 (77.5%)	128 (78.5%)
Male	9 (20.0%)	8 (20.5%)	9 (23.1%)	9 (22.5%)	35 (21.5%)
BMI					
* M* (SD)	23.1 (3.93)	21.7 (2.79)	22.5 (4.93)	22.6 (3.65)	22.5 (3.90)
Median [min, max]	22.3 [17.9, 37.1]	21.5 [16.5, 28.4]	21.3 [17.3, 47.9]	21.6 [18.2, 35.1]	21.6 [16.5, 47.9]
Smoking status					
Non-smoker	44 (97.8%)	35 (89.7%)	34 (87.2%)	35 (87.5%)	148 (90.8%)
Smoker	1 (2.2%)	4 (10.3%)	5 (12.8%)	5 (12.5%)	15 (9.2%)
Cigarettes per day in smokers					
* M* (SD)	0	6.75 (5.68)	2.60 (2.51)	2.20 (3.03)	3.40 (4.01)
Median [min, max]	0 [0, 0]	5.00 [2.00, 15.0]	3.00 [0, 5.00]	0 [0, 6.00]	3.00 [0, 15.0]
Hormonal Contraceptives in Females					
No	31 (86.1%)	23 (74.2%)	20 (66.7%)	20 (64.5%)	94 (73.4%)
Yes	5 (13.9%)	8 (25.8%)	10 (33.3%)	11 (35.5%)	34 (26.6%)
Current menstruation in females					
No	27 (75.0%)	25 (83.3%)	22 (73.3%)	26 (83.9%)	100 (78.7%)
Yes	9 (25.0%)	5 (16.7%)	8 (26.7%)	5 (16.1%)	27 (21.3%)
Shift work					
No	38 (84.4%)	33 (84.6%)	37 (94.9%)	36 (90.0%)	144 (88.3%)
Yes	7 (15.6%)	6 (15.4%)	2 (5.1%)	4 (10.0%)	19 (11.7%)
Time awake (in hours)					
* M* (SD)	6.42 (2.18)	5.66 (2.28)	6.37 (2.26)	6.62 (2.30)	6.28 (2.26)
Median [min, max]	6.50 [1.50, 13.3]	5.50 [1.50, 10.3]	5.90 [3.00, 12.0]	7.00 [2.50, 11.3]	6.10 [1.50, 13.3]
Regular mindfulness practice					
No	29 (64.4%)	25 (64.1%)	28 (71.8%)	27 (67.5%)	109 (66.9%)
Yes	16 (35.6%)	14 (35.9%)	11 (28.2%)	13 (32.5%)	54 (33.1%)
Room temperature					
* M* (SD)	27.4 (2.73)	26.0 (2.78)	27.5 (3.16)	26.3 (2.92)	26.7 (2.96)
Median [min, max]	27.9 [19.1, 30.0]	26.7 [19.1, 30.1]	29.0 [19.9, 30.5]	26.7 [19.9, 30.2]	27.6 [19.1, 30.5]
Log-transformed sCort Baseline (ng/mL)					
* M* (SD)	1.62 (0.43)	1.64 (0.53)	1.57 (0.44)	1.59 (0.47)	1.61 (0.46)
Median [min, max]	1.64 [0.64, 2.48]	1.65 [0.55, 2.71]	1.55 [0.68, 2.82]	1.57 [0.83, 2.60]	1.58 [0.55, 2.82]

sCort Baseline levels are log-transformed (ln) and do not differ significantly across conditions.

### Outcomes

Saliva samples were obtained using specialized collection tubes (SaliCab®, RE69985, IBL, Hamburg, Germany) to assess salivary cortisol (sCort) concentrations. Study participants were guided to passively drool the saliva and to allow it to flow naturally through a plastic straw into the collection vessel. Following collection, the samples were preserved at −80 °C until further processing. The quantification of sCort concentrations, expressed in (ng/mL), was performed using a commercial immunoassay kit (RE52611; IBL, Hamburg, Germany) in accordance with the provided protocol. All biochemical analyses were conducted at the Institute of Medical Psychology’s stress biomarker facility in Heidelberg. To enhance precision, 20% of the samples underwent duplicate analysis, with the resulting mean values used in subsequent statistical evaluations. The intra-assay coefficient of variation (CV) was 4.63%. The inter-assay CV was 3.79%. To account for potential confounding influences on salivary cortisol levels, sensitivity analyses were conducted including variables such as age, sex, body mass index (BMI), smoking status and number of cigarettes per day, use of hormonal contraceptives and current menstruation in females, shift work, time awake prior to testing, regular mindfulness practice, and room temperature^cf.^ ^46,78^.

The subjective experience of stress was assessed using visual analog scales (VAS) based on the methodology of Gaab et al.^79^, which in turn goes back to earlier work by Kirschbaum et al.^80^. Building on the approach of Gaab et al.^79^, two VAS were used to assess retrospective primary appraisal of acute subjective stress following stress induction, focusing specifically on perceived stressfulness and challenge as the most relevant dimensions for the present study. The remaining two items concern perceptions of control over the situation, which were not central to our research aims and not conceptually appropriate in the context of a standardized script. For this reason, they were not included. The assessment took place immediately after the second simulated medical consultation, with participants rating their experience on a scale ranging from 0 to 100. The scale anchors were labeled as “not at all” (0) and “very much” (100). Participants rated the following two items: “The past situation was stressful for me” and “I found the past situation to be a challenge”. The mean score of these two items was calculated to yield a composite measure of retrospective primary appraisal, with higher values on this measure indicating a more intense subjective stress experience. In the following, this composite score is referred to as subjective stress.

The questions assessing memory retrieval were designed based on the information provided to participants during the simulated conversations (“e.g. “Based on which symptoms can the onset of the disease be identified?”) and on what they could perceive from their conversational environment. In total, 6 open-ended questions, 7 closed-ended questions, and 3 questions about the visual environment were administered. For visual memory questions, it was ensured that the same information was presented for each condition, e.g., also by showing a screenshot of the avatar in the chatbot condition, and by standardizing the appearance of the human and AI physician. All participants were given the same questions, independent of the condition. A detailed overview of the questions is provided in Supplementary Material D). Each answer to the question was coded as correct (=1) or incorrect (=0). For each question type (open-ended, closed-ended, and visual perception), mean scores were calculated.

To evaluate the credibility of the situation, we included a self-generated question: “Were there any moments during the conversation when you had the impression that you might actually have Huntington’s disease?” Participants responded using a VAS ranging from 0 (“not at all”) to 100 (“very strongly”). This item served as a manipulation check to evaluate participants’ immersion in the scenario and was administered following the interviews. In addition, an item was interpreted to validate the perception of the simulation (“In your opinion, was the conversation realistic enough to resemble a non-simulated physician–patient interaction?”, VAS ranging from 0 (“not at all”) to 100 (“very strongly”)). This exploratory validation analysis is reported in the Supplementary Materials F).

### Statistical analyses

For all sCort analyses, *N*_*sCort*_ sample was used. All other analyses not containing sCort variables used *N*_*total*_. Group differences for all four consultation modalities were analyzed with analyses of variance (ANOVAs), except for sCort concentrations, which were analyzed using a longitudinal linear mixed-effects model fitted to log-transformed sCort values. Post hoc tests were conducted following significant ANOVA results to explore pairwise group differences. The model included fixed effects for time, condition, and their interaction, and random intercepts and slopes for time per participant to account for individual baseline levels and change trajectories. This approach accounts for within-person dependencies over time. The model was estimated using restricted maximum likelihood (REML) in R (version 4.4.3), using the lme4^81^ and lmerTest^82^ packages. Degrees of freedom for inferential statistics were approximated using the Satterthwaite method. For all parametric analyses, assumptions of normality and homogeneity of variances were assessed prior to hypothesis testing. Normality was evaluated using Shapiro-Wilk tests and inspection of residual distributions, and homogeneity of variances was assessed using Bartlett’s tests. In cases where assumptions for parametric testing were violated, non-parametric alternatives were applied. Potentially influential observations were assessed using Cook’s distance, and sensitivity analyses excluding identified outliers were conducted to assess robustness.

As an exploratory step, the conditions were additionally compared by grouping them into human-based agent (in-person and video visit) and AI*-based agent (AI* chatbot and AI* avatar) to test for the effect of interaction partner agency. Differences in subjective stress, sCort concentrations, and perceived credibility of the situation between these groups were analyzed. Depending on whether the assumptions for parametric testing were met, either t-tests or Wilcoxon rank tests were conducted to assess these differences.

Three regression models were constructed to predict memory retrieval, each corresponding to a specific memory assessment (open format, closed format, visual). The independent variables in these regressions included conditions, the Area under the Curve with respect to increase (AUCi)^83^ of sCort, and their interactions.

Two multiple linear regression models were calculated to assess how perceived credibility of the situation, in combination with conditions, affects subjective stress and sCort (AUCi).

Statistical significance for all analyses was defined as *p* < .05.

### Ethics declaration

Ethical approval was granted by the Ethics Committee of the Faculty of Medicine at Heidelberg University (Ethics approval number: S-067/2022), and all participants provided informed consent in accordance with the Declaration of Helsinki.

## Supplementary information

Supplementary Materials

## Data Availability

The datasets used and/or analyzed during the current study are available from the corresponding author on reasonable request.

## Code availability

All statistical analyses were conducted using R version 4.4.3 (2025-02-28). R codes are not publicly available but may be made available from the corresponding author on reasonable request.
