# Supplementary material for: Perceptions of simulated artificial intelligence in medical consultations: associations with stress, memory, and perceived credibility
Source: NPJ Digit Med. 2026 Jul 29;9:586. doi: 10.1038/s41746-026-03022-5 (PMC13421662; doi:10.1038/s41746-026-03022-5)
Supplement: Supplementary file 1 — Supplementary Materials [file 41746_2026_3022_MOESM1_ESM.pdf]

Supplementary Materials

Manuscript: Perceptions of Simulated Artificial Intelligence in Medical Consultations: Associations with Stress, Memory, and Perceived Credibility

Carlotta J. Mayer [1,2] , Tristan L. Swysen [2,3], Tobias R. Kurz [2,3], Tobias I. Stephan [2,3], Daniela Geisel [4], Elena S. Doll [2,3], Nassim Mahal [2,3], Seraina P. Lerch [5], Sebastian Sailer [6], Christian P. Schaaf [6,7], Christian J. Merz [8], Torsten Wüstenberg [9], Johannes C. Ehrenthal [10], Steffen Walter [4], Julia Mahal [2,3 *], Beate Ditzen [2,3,7,11 *]

[1] Laboratory for Clinical Neuropsychology, Department of Psychology, Heidelberg University, Heidelberg, Germany

[2] Institute of Medical Psychology, Heidelberg University Hospital, Heidelberg, Germany

[3] Heidelberg University, Heidelberg, Germany

[4] Clinic of Psychosomatic and Psychotherapy, Department of Medical Psychology, University Hospital Ulm, Germany

[5] Department of Medical Psychology, University Medicine Greifswald, Greifswald, Germany

[6] Institute of Human Genetics, Heidelberg University, Medical Faculty Heidelberg, Heidelberg, Germany

[7] German Centre for Mental Health, Partner Site Heidelberg-Mannheim-Ulm, Mannheim, Germany

[8] Department of Cognitive Psychology, Institute of Cognitive Neuroscience, Faculty of Psychology, Ruhr University Bochum, Bochum, Germany

[9] Core Facility for Neurocognitive Systems Research (CNSR), Heidelberg University, Heidelberg, Germany

[10] Department of Psychology, University of Cologne, Cologne, Germany

[11] Clinical Biopsychology and Psychotherapy, Department of Psychology, University of Zurich, Zurich, Switzerland

[*] These authors contributed equally.

A) Ethical Management Protocol

Receiving a simulated serious medical diagnosis can be emotionally intense. Therefore, a multi-stage protocol was implemented to protect the psychological well-being of all participants.

Pre-Encounter Safeguards
Before the experiment, participants received detailed written and verbal information. The researchers explicitly emphasized that the upcoming consultation is a purely fictional simulation for research purposes and that no real medical diagnostics or health evaluations take place. Participation is entirely voluntary. Participants can withdraw at any point without giving reasons and without any negative consequences.

During the Encounter
The communication script was strictly standardized across all conditions. However, researchers continuously monitored participants via a live-stream video setup. This allowed the team to immediately detect any signs of severe acute distress.

Post-Encounter Safeguards and Debriefing
Immediately after the consultation, the experimenters conducted a debriefing session with each participant: The experimenters verbally reinforced the simulation framework. This ensured a clear boundary between the experiment and reality. Participants had sufficient time to ask questions, express concerns, and emotionally process the situation. Before departure, the experimenters carefully ensured that each participant felt comfortable and well enough to leave. Upon request or if a need for follow-up discussion arose, the team provided business cards with the study team's contact details and shared the contact information of the institute's outpatient clinic. This ensured that immediate psychological support was accessible in the highly unlikely event of delayed distress.

B) Script for the standardized and simulated medical consultation

Preamble

The following text represents the standardized consultation script that served as the foundation for all four experimental conditions in this study. It was developed to ensure that each simulated medical consultation followed an identical structure and content flow, thereby allowing systematic comparison across modalities. The script outlines the full course of the simulated genetic counseling scenario, including the welcoming phase, the explanation of the screening test and Huntington’s disease, the joint creation of a family pedigree, and the delivery of a positive genetic test result.

For the two AI*-based consultation modalities (chatbot and avatar), the same content was implemented using a **Wizard-of-Oz (WOZ)** procedure. In these conditions, participants were informed that they were interacting with an autonomous AI* physician, while in reality a trained operator controlled the interaction in real time. To enable smooth and natural exchanges without noticeable delays, the operator required access to pre-prepared response elements. Therefore, this master script was used as the basis for generating a comprehensive set of **text modules** representing all statements and explanations contained in the human-physician version.

To facilitate flexible and context-sensitive communication, longer text passages from this script were segmented into smaller units that could be combined or selected according to the course of the conversation. In addition, a limited set of short **filler phrases** was prepared to allow the operator to respond promptly to participants’ spontaneous remarks or questions while maintaining the flow of dialogue. Examples of such fillers include brief acknowledgments (e.g., “I understand,” “Exactly,” “Please give me a moment”) and transitional expressions to manage pacing. This modular adaptation was essential for maintaining the illusion of an autonomous AI system, as it minimized response latency and ensured consistency across participants.

The resulting materials thus provided a unified framework for all four conditions (human in-person, human video visit, AI* chatbot, and AI* avatar) while preserving the standardized informational content and emotional tone of the simulated medical consultation.

Table S1. Script used for the Standardized Simulation of the Medical Consultation

**Part 1**		
**Section 1: Welcoming**		
Good day, my name is Dr. Komm, and I'm responsible for conducting the initial consultations in genetic counseling here. I'm glad you were able to make it today. This is an important appointment.<br>We've created an anonymized file for you, and you were given an identification code for your saliva sample. Could you please tell me this code so that I can verify it?		
Thank you. May I also ask your current age?		
**Section 2: Screening Test**		
Good, thank you. I now have everything I need here. Perhaps before we continue: Has anyone explained to you what kind of screening test your saliva sample was used for?		
	Participant doesn't know	
No problem. I'd like to begin by explaining what this test is about. At the moment, we are evaluating a new rapid screening method for certain hereditary diseases. Hereditary diseases are conditions that develop because of specific changes — or errors — in a person's genetic material, that is, in their DNA.<br><br>Some hereditary diseases appear very early in childhood, while many others develop only later in life. That means people are born with this genetic change and may carry it for decades before any symptoms appear.		
With the rapid tests we are currently developing here at the University Hospital, this may change in the future. These tests allow for early screening so that people at risk can be identified before they become ill.<br><br>Up to now, identifying such risks requires a detailed DNA analysis — a process that is expensive and therefore usually reserved for situations where there is a so-called initial suspicion that something in the genes might not be quite right.		

Typically, such an initial suspicion arises when a hereditary disease is already known in the family or when the person shows symptoms consistent with it. With the new screening tests, it's different. We can now test otherwise healthy individuals at low cost and identify whether there is an increased probability for a particular hereditary disease.		
It's important to understand that when we talk about the results of a screening test, we're referring to an increased probability, not a confirmed diagnosis. This does not necessarily mean that genetic material is defective — but it provides an initial indication that leads us to perform a more precise genetic analysis.		
Does that make sense to you so far?		
	Participant responds	
I have here the result of your screening test. In your case, we found an increased probability for a condition called Huntington's disease. This is a serious illness, which is why I've invited you to this genetic counseling session today.		
**Section 3: Explanation about Huntington's Disease**		
What do you already know about Huntington's disease?		
	Participant does not know the disease	That's completely fine — let me give you a brief overview.
	Participant knows something about the disease	That's all correct.
	Participant has some misconceptions	You're right about *[specific part].* However, I'd like to clarify that *[correction].*
Huntington's disease is a neurodegenerative condition, meaning that over time, certain brain cells gradually die off.		
There are three main areas in which the disease becomes especially noticeable:<br><br>- **Motor function** — movement and coordination,<br>- **Cognitive abilities** — memory and thinking, and<br>- **Emotional and psychological well-being** — which can also affect a person's personality.		

The motor symptoms often include movement disorders such as slowed or jerky movements, trembling, clumsiness, or restlessness. In the later stages, some patients may lose the ability to walk.		
Cognitive performance also declines — similar to what we see in the early stages of dementia. Symptoms can include forgetfulness, slower thinking, and decreased concentration or distractibility.		
At the beginning of the disease, the first changes are often psychological — irritability, restlessness, or subtle changes in behavior. The person's personality can alter significantly; for instance, someone might begin to have sudden outbursts of anger or, conversely, withdraw completely, becoming depressed or even psychotic.		
Once the symptoms begin, they unfortunately do not go away — instead, they gradually worsen. In the advanced stages, most patients lose the ability to move, swallow, or speak, and experience a significant decline in cognitive function. Most people at that stage require full-time care.		
There is currently no cure for Huntington's disease, and it is, sadly, a progressive and ultimately fatal condition. Most affected individuals die from respiratory failure or pneumonia, often because swallowing difficulties cause food or liquid to enter the lungs.		
While we can't stop progression, certain symptoms can be managed or alleviated with medication or supportive therapy.<br>Typically, the disease begins around the age of 45, and the symptoms at first may be so mild that they're easily overlooked. From there, the disease advances gradually over time, and most patients live for about 15 years after symptom onset.<br>Because Huntington's is an inherited condition, a person who carries the mutation has a 50% chance of passing it on to each child.		
I know that's a lot of information about a very serious condition. Is everything clear so far, or do you have any questions at this point?		
	[Pause to allow for questions; address them if appropriate, without advancing too far ahead in the conversation.]	

**Section 4: Pedigree**		
Good. Next, I'd like to create your family tree together with you. I assume that no one in your family has ever been diagnosed with Huntington's disease — is that correct?		
	Participant responds (all responded no)	
Even so, it's possible that someone in your family may have shown symptoms that fit Huntington's disease, but which were never identified as such. In the past, medical understanding wasn't always advanced enough to recognize hereditary diseases like this one clearly. But it's also possible that the disease has not yet appeared in your family and that you may be the first affected person.		
Do you have any siblings? If so, how many brothers and how many sisters?		
	Participant responds, operator provides adequate pedigree template	
Then this is your family tree. Circles represent female family members, and squares represent male family members. We'll start from the top — your grandparents, generation one — and move down to your parents, then to you (and your siblings), generation three. I'll ask you a few questions about any possible findings or symptoms.		
First question: Are all the family members listed here still living?		
	Participant responds "yes"	*[Operator continues with next line]*
	Participant responds "no"	Do you know the cause of death for those who have passed away?
[For each of the four grandparents and both parents one by one.] Regarding your [mother's father], have you noticed or do you know of any symptoms that might fit with Huntington's disease?<br>- Motor function: tremors, twitching, being bedridden in old age<br>- Cognition: concentration problems, forgetfulness<br>- Psychological state: depression or any other mental illness?<br>- Personality: changes such as sudden outbursts of anger or sudden apathy?		
Are there any symptoms or abnormalities among your siblings?		

- Motor function: tremors, twitching, being bedridden in old age<br>- Cognition: concentration problems, forgetfulness<br>- Psychological state: depression or any other mental illness?<br>- Personality: changes such as sudden outbursts of anger or sudden apathy?		
Have you noticed any early symptoms in yourself — for instance, shaky hands, problems with concentration or memory, inner restlessness, or clumsiness?		
	If participant reported none of the symptoms in family members	Based on your family and personal symptoms, there are no current signs of the disease. However, that doesn't necessarily mean anything — we'll confirm this through genetic analysis.
	If participant reported some of the symptoms in family members	Based on your family or personal history, there are some indications that require clarification — we'll confirm this through genetic analysis.
Do you have any further questions right now?		
	Participant says "yes"	That's a good question — we'll go over it in detail once we have your results, as that will tell us which direction we'll need to take next.
	Participant says "no"	*[Operator continues with next line]*
**Section 5: Genetic Analysis**		
I'll now step out briefly to have your genetic analysis processed in the lab.<br>In the meantime, my assistant will take another saliva sample from you for the archive so that we can repeat or verify the test later if necessary.<br>I'll return in about five to ten minutes once the results are ready.		
**Part 2**		
**Section 6: Communication of bad news**		
I've now received the results of your personal genetic analysis. Before we discuss them — have any questions come up for you in the meantime?		
	Participant asks a question	*[Operator responds briefly]*

	Participant does not ask a question	*[Operator continues with next line]*
How are you feeling right now?		
	Participant answers “nervous” or “bad”	I can completely understand that.
I’ll get straight to the point: unfortunately, I have some very bad news for you.<br><br>The genetic analysis has identified the mutation in your sample.		
	[Optional addition if the patient is under 35:]	In your case, it’s a severe variant of the mutation, which means that the first symptoms are likely to appear earlier than usual — possibly much earlier than the mid-forties.
	[If the patient asks at what age, add:]	Roughly speaking, we’d expect the first symptoms around [current age + 5 years].
I’m very sorry.		
Is there a particular question, thought, or feeling going through your mind right now that you’d like to share with me?		
	Participant expresses a question, thought, or emotion	*[Operator responds appropriately based on the patient’s comment, then continues in the next line — or if the patient says nothing, continue directly.]*
	Participant does not say anything	*[Operator continues directly.]*
How are you coping with this news?		
	Participant responds	
What does this result mean for you and your life going forward?		
	Participant responds	*[Operator continues directly.]*
	Participant does not respond	Are you feeling afraid or uncertain about what this means for you? Or are you experiencing this more rationally at the moment?
Who in your life can you talk to about this — who would you consider a trusted person?		

	Participant responds	
How do you think this person will react?		
	Participant responds	
What kind of work do you currently do?		
	Participant responds	
Until the disease begins to manifest, you won’t experience any specific limitations in your daily life or work. However, given your genetic result, we must expect that symptoms may appear relatively soon.		
In the early phase, most people are still able to manage daily activities and continue working, but the condition can progress noticeably within a few years. That’s why it’s advisable to start preparing now — both practically and emotionally.		
Unfortunately, there’s no medication available that can prevent or delay the onset of the disease. However, we know that an unhealthy lifestyle — including smoking, alcohol, or other substances — can accelerate its onset.		
Perhaps the most difficult factor for most people is stress. Chronic stress is one of the most important triggers for an earlier disease onset.<br><br>It’s therefore very important to take good care of yourself, avoid unnecessary strain, and lead as healthy a life as possible.		
If you don’t have any more questions for today, I’ll now prepare your report. I’ll also give you referrals to several specialists whom you should see soon and then visit regularly.		
One will be a referral to a neurologist, and another to a psychiatrist.<br>Since you’re currently almost symptom-free, it’s best to see these specialists early so they can recognize and monitor the development or onset of the disease as soon as possible. There are also several counseling services that can assist you — including practical matters such as life insurance, occupational disability, or long-term care.<br>And of course, they can support you in coping with this difficult diagnosis now and later, once the disease progresses. I’ll prepare the medical letters for you now and also bring you the information materials.		
		[For chatbot /avatar condition: “I’ll make these available for download here shortly.”]

All right. My assistant will come by once more to take an additional saliva sample for the archive, so that we can repeat or verify results later if necessary.<br>I'll be back shortly with the letters / the letters will be ready for download soon, and then you'll be able to go home.		
**Optional responses and variations**		
Please give me a moment.		
Thank you.		
Is that clear so far?		
Do you have any questions at this point?		
Was that understandable for you, or would you like me to explain further?		
Please bear with me for a moment.		
Okay.		
I understand.		
Yes.		
Exactly.		
Good.		
I'm sorry, I interrupted you — what were you about to say?		
That's an important question; we'll come back to it shortly.		
We can ease certain symptoms, but we cannot stop the disease from progressing.		
Unfortunately, no medication can delay onset.		
The probability of actually developing the disease after a positive screening test is about one-third.		

C) Screenshots of AI*-physician Conditions

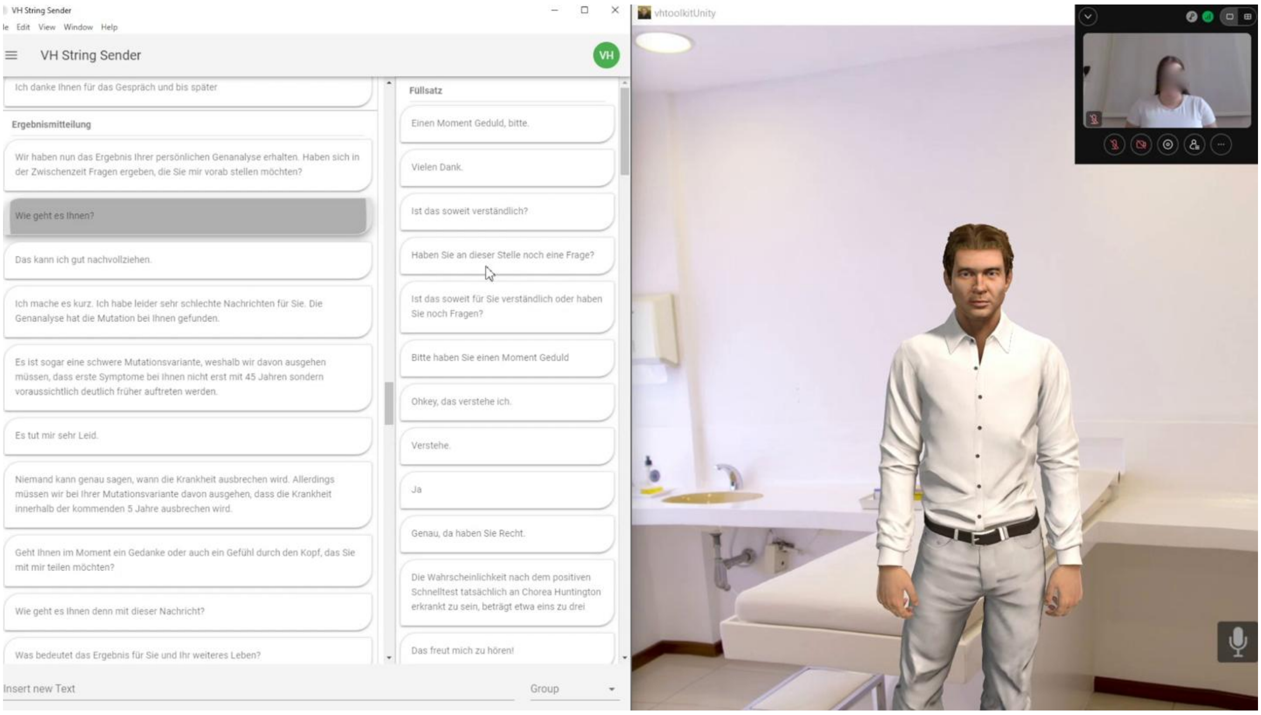

Figure S1. Operator's view of the AI interface

Figure S1 shows the interface of the AI* avatar from the operator’s view. This includes the operational interface on the left side of the screen and the output of the avatar representation on the right side of the screen (adaption from the Virtual Human Toolkit). This output was displayed to participants on a screen.

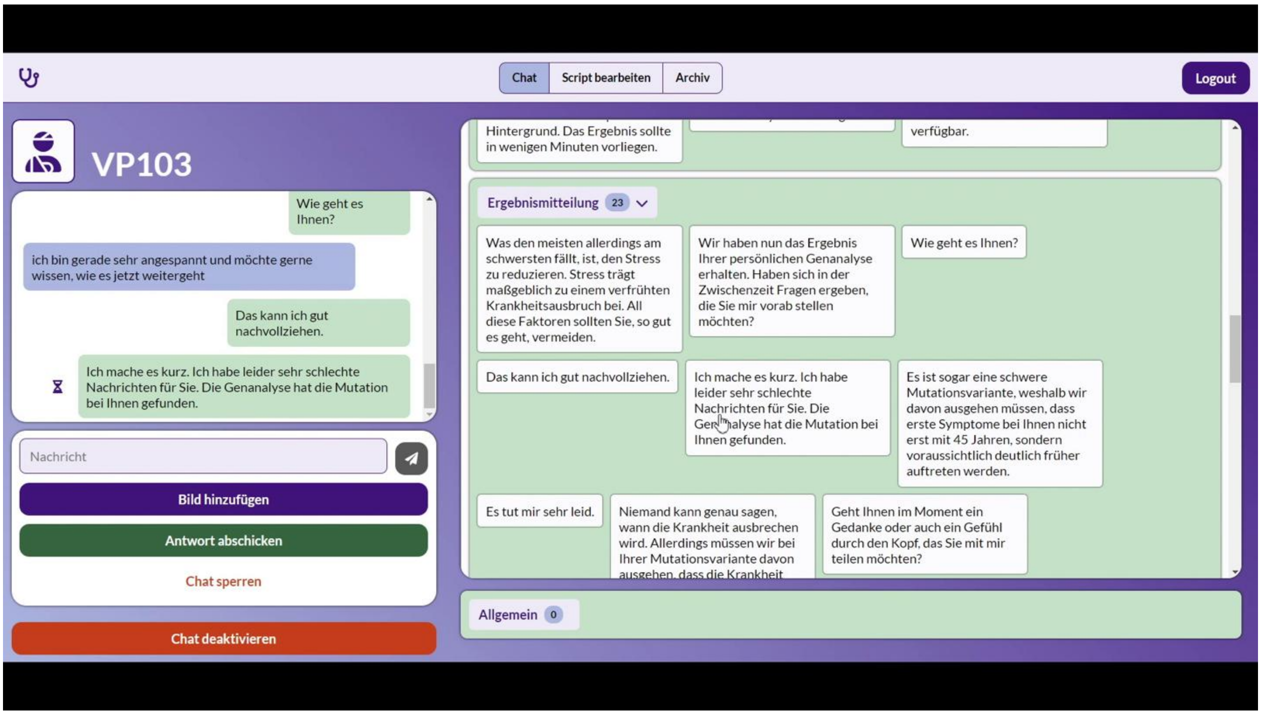

Figure S2. Operator's view of the AI chatbot interface

Figure S2 shows the interface of the AI*chatbot from the operator’s view.

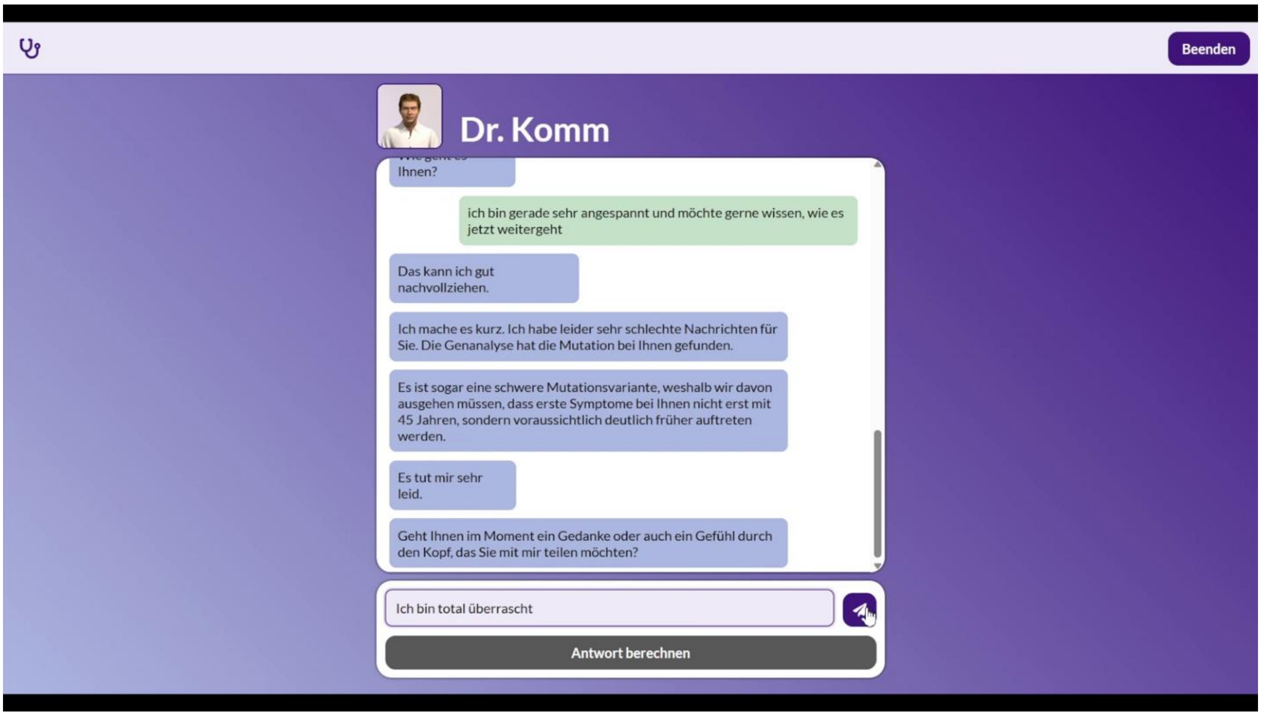

Figure S3. Participant's view of the AI chatbot interface

Figure S3 shows the interface of the AI*chatbot from the participant‘s view.

D) Questions on Memory Retrieval

Table S2. Questions on Memory Retrieval

Question Type	Item
Open format	What psychological and cognitive symptoms can occur in Huntington's disease? (5 max.)<br><br>What physical symptoms can occur in Huntington's disease? (5 max.)<br><br>By which symptoms can the beginning of the disease be identified? (5 max.)<br><br>What symptoms occur in the late stage of the disease? (5 max.)<br><br>What is the average survival time after the onset of the first symptoms?<br><br>What do most patients die from?<br><br>What advantages does the novel rapid test offer compared to previous analysis methods for Huntington's disease?<br><br>How many of the individuals classified as at risk for Huntington's disease by the new screening method are actually carriers of the mutation? (1 out of _ individuals)<br><br>If the genetic mutation is present: What is the probability of passing the genetic mutation on to children? (_ Percent)
Closed format	At what age do symptoms of Huntington's disease typically begin? (35/40/45/50/55/I don't remember)<br><br>Which statement about the treatment options for Huntington's disease is correct? (There is no cure; treatment is limited to symptomatic management./ If Huntington's disease is detected early (before age 20), a complete cure is possible. /If Huntington's disease is detected and treated early (before age 25), patients' life expectancy is not limited./If Huntington's disease is detected and treated early (before age 25), the likelihood of a severe disease course decreases./If Huntington's disease is treated promptly after the first symptoms appear, the development of severe symptoms can be prevented. /I don't remember)<br><br>To which type of diseases does Huntington's chorea belong? (Blood clotting disorder /Neurodegenerative disease of the brain /Demyelinating disease /Metabolic disease /Chronic inflammatory disease /I don't remember)<br><br>In the pedigree analysis, female individuals were represented by which symbol? (Square /Circle/ Triangle/ Trapezoid / Hexagon/ I don't remember)
Visual Perception	What was the name of the physician you were just interacting with?

	Was the physician you were interacting with wearing a white coat? (Yes/No/I don’t remember) (For this question, answering “yes” was coded as correct for participants in the human-physician conditions, and answering “no” was coded as correct for participants in the AI-physician conditions.)<br><br>Did the physician you were interacting with have brown hair? (Yes/No/I don’t remember)<br><br>Did the physician you were interacting with have a stethoscope on? (A stethoscope can be placed on the body to listen to, for example, the heartbeat.)

E) Sensitivity Analysis of human-physician versus AI*-physician conditions and cortisol concentrations excluding all outliers

Table S3. Sensitivity Analysis for outliers

Predictor	*b*	*t*(*df*)	*p*
(Intercept)	1.74	26.26 (106.00)	<.001 ***
time	-.08	-5.82 (106.00)	<.001 ***
AI*agent	-.11	-1.20 (106.00)	.233
time * AI*agent	-.04	-2.35 (106.00)	.020 *

Note. *** $p < .001$, ** $p < .01$, * $p < .05$

F) Exploratory analysis of perceived realism of the interaction

Item: “In your opinion, was the conversation realistic enough to resemble a non-simulated physician–patient interaction?”, VAS from 0 – 100

Table S4. Perceived realism of the interaction across consultation modalities

	n	*M*	*SD*
Human in-person	45	73.44	25.98
Human video visit	39	74.72	23.51
AI*chatbot	39	65.10	27.34
AI*avatar	40	46.73	31.44
ANOVA	$F(3,159) = 9.12$, $p < .001$		
